# Integrated transcriptomic and hormonal analysis suggests a trade-off between salt/drought tolerance and thermotolerance in *Brassica rapa*

**DOI:** 10.3389/fpls.2026.1868194

**Published:** 2026-07-27

**Authors:** Mei Zheng, Peirong Li, Xiaoyun Xin, Weihong Wang, Xiuyun Zhao, Deshuang Zhang, Yangjun Yu, Jiao Wang, Fenglan Zhang, Tongbing Su, Shuancang Yu

**Affiliations:** 1 State Key Laboratory of Vegetable Biobreeding, Beijing Vegetable Research Center (BVRC), Beijing Academy of Agriculture and Forestry Science (BAAFS), Beijing, China; 2 Key Laboratory of Biology and Genetic Improvement of Horticultural Crops (North China), Ministry of Agriculture, Beijing, China; 3 Beijing Key Laboratory of Crop Molecular Design and Intelligent Breeding, Beijing, China; 4 National Engineering Research Center for Vegetables, Beijing, China

**Keywords:** anthocyanin, *Brassica rapa*, plant hormones, plant stress response, transcriptome

## Abstract

**Introduction:**

*Brassica rapa* plants often face combined or sequential abiotic stresses, but the potential negative association between salt/drought tolerance and thermotolerance remains poorly understood. Most studies have focused on individual stress responses, leaving the regulatory networks that may constrain broad-spectrum resilience largely unexplored.

**Methods:**

Here, we propose a hormone- and anthocyanin-centered framework for understanding this apparent negative association in *B. rapa*, based on integrated physiological, transcriptomic, hormonal and metabolic analyses across diverse inbred lines.

**Results:**

Physiological characterization of 11 lines revealed an apparent negative association (salt vs. heat: r = –0.555; drought vs. heat: r = –0.339). Time-resolved transcriptomics uncovered stress-specific temporal patterns: a triphasic response under salt stress, a 'rapid response–readjustment–reactivation' pattern under drought, and a biphasic mechanism under heat stress. Hormonal profiling identified ABA and ethylene biosynthesis-related metabolites as correlates of osmotic adaptation. The chalcone synthase gene *BraA10g024990.3C (CHS)* showed genotype- and stress-specific expression and correlated with anthocyanin accumulation. Exogenous hormone treatments indicated that ABA and ethylene induce, while GA represses, *CHS*-mediated anthocyanin production.

**Discussion:**

Despite the correlative nature of these data, this study provides a candidate regulatory axis and suggests that hormone-directed anthocyanin metabolism may contribute to the negative association between osmotic tolerance and thermotolerance in *B. rapa*, offering targets for breeding multi-stress-resilient crops.

## 1 Introduction

*Brassica rapa* L. (*B. rapa*), is a globally significant vegetable crop and a cornerstone species within the economically and nutritionally vital *Brassicaceae* family. However, its widespread cultivation is increasingly threatened by the escalating prevalence and intensity of abiotic stresses, particularly salinity (Irulappan et al., 2023; Bano et al., 2022), drought (Johnson and Rieseberg, 2022; Muthusamy et al., 2020; Zhang et al., 2023), and high-temperature (heat) stress (Quan et al., 2023; Zhang et al., 2022; Wang et al., 2024; Cao et al., 2023). These environmental constraints not only occur independently but frequently manifest in combination or succession within dynamic agricultural ecosystems, imposing complex and often antagonistic demands on plant adaptation (Zandalinas and Mittler, 2022). The concurrent or sequential occurrence of such stresses poses a formidable challenge for sustainable crop productivity, especially under climate change, where the frequency and severity of these events are projected to increase (Reynolds et al., 2015).

Decades of research have yielded substantial insights into the physiological and molecular mechanisms underlying plant responses to individual abiotic stresses. For example, osmotic stress tolerance, crucial for surviving salinity and drought, typically involves abscisic acid (ABA)-mediated stomatal closure (Mishra et al., 2006; Chen et al., 2020; Liu et al., 2022), accumulation of compatible osmolytes (Yang and Guo, 2017; Wang et al., 2019), and enhanced antioxidant defense systems (Laxa et al., 2019). In contrast, thermotolerance often relies on rapid induction of heat shock proteins (HSPs) (Mondal and Singh, 2023), maintenance of membrane fluidity (Higashi and Saito, 2019), and activation of specific transcriptional networks involving heat shock factors (HSFs) (Ohama et al., 2017). While these pathways are reasonably well-characterized in isolation, a less understood aspect is how plants prioritize resource allocation and signaling pathways when confronted with multiple, potentially conflicting stresses. This phenomenon, often referred to as a physiological or molecular ‘trade-off,’ implies that robust adaptation to one stressor may come at the expense of tolerance to another. Evidence suggests that the molecular machinery optimized for osmotic stress (e.g., high ABA signaling, energy-intensive osmolyte synthesis) might inherently conflict with the requirements for effective heat dissipation and protein stability maintenance under high temperature, potentially constraining broad-spectrum resilience (Xu et al., 2025; Kan et al., 2023; Yu and Li, 2024).

Plant hormone signaling networks, acting as central orchestrators of stress adaptation, are prime candidates for mediating such negative associations. Key hormones including ABA, jasmonic acid (JA), cytokinins (CTK), brassinosteroids (BR), ethylene (ETH), and gibberellins (GA) dynamically interact to fine-tune stress responses (Verma et al., 2016; Waadt et al., 2022). While ABA is well established as a master regulator of drought and salt responses, and ethylene plays roles in various stress adaptations, their specific interplay with hormones like CTK and BR (implicated in growth and potentially heat responses) remains poorly resolved in the context of cross-stress tolerance trade-offs. Furthermore, secondary metabolites, including flavonoids and anthocyanins, contribute to stress mitigation through antioxidant activity (Di Ferdinando et al., 2012), UV protection (Ferreyra et al., 2021), and osmoregulation (Dabravolski and Isayenkov, 2023). Their biosynthesis is tightly regulated by hormonal and environmental cues (Zheng et al., 2025;Patil and Sharma, 2024), but how their production is differentially prioritized under contrasting stresses, and whether this contributes to observed trade-offs, requires deeper investigation, particularly in *B. rapa*.

In this study, we employed an integrated physiological, transcriptomic, hormonal and metabolic approach across a panel of *B. rapa* inbred lines with contrasting stress responses. We hypothesized that an apparent negative association between osmotic tolerance and thermotolerance exists at the molecular level, driven by distinct hormonal signatures and metabolic regulation. Our objectives were: (1) to physiologically characterize the negative association between salt/drought tolerance and thermotolerance; (2) to delineate the temporal transcriptional programs underlying salt, drought and heat responses; (3) to profile hormonal changes associated with stress-specific adaptation; and (4) to identify candidate genes and metabolic pathways, particularly in anthocyanin biosynthesis, that may contribute to this association. Through time-course transcriptome sequencing, targeted hormone quantification, and exogenous hormone treatments, we identified a candidate regulatory axis linking hormone-directed anthocyanin metabolism to the observed negative association. These findings provide molecular insights and potential targets for future breeding of multi-stress-resilient *B. rapa*.

## 2 Results

### 2.1 Physiological characterization reveals an apparent negative association between salt/drought tolerance and thermotolerance

Screening of 11 *B. rapa* inbred lines revealed an apparent negative association between salt/drought tolerance and thermotolerance (**Supplementary Figures 1A–C**). To quantify this relationship, we performed Pearson correlation analysis using wilting rates under salt, drought and heat stress. A strong positive correlation was observed between salt and drought stress responses (r = 0.921) (**Supplementary Figure 1D**), indicating that lines with higher salt tolerance also tended to possess higher drought tolerance. In contrast, negative correlations were detected between salt and heat stress (r = –0.555) and between drought and heat stress (r = –0.339) (**Supplementary Figure 1D**), supporting the existence of a negative association between osmotic (salt/drought) tolerance and thermotolerance. Furthermore, based on the integrated tolerance scores (salt, drought and heat tolerance), the 11 lines were clustered into two distinct groups. Group I (thermotolerant: 240, 16A-1, JL, R0–18 and WJ006) exhibited high heat tolerance but low salt/drought tolerance, whereas Group II (salt/drought tolerant: 32, 37, 49, 123, 129 and 16N-1132) showed the opposite pattern (**Supplementary Figure 1E**). Within each group, the stress tolerance traits were highly consistent, further corroborating the divergent adaptive strategies among *B. rapa* germplasm.

**Figure 1:**
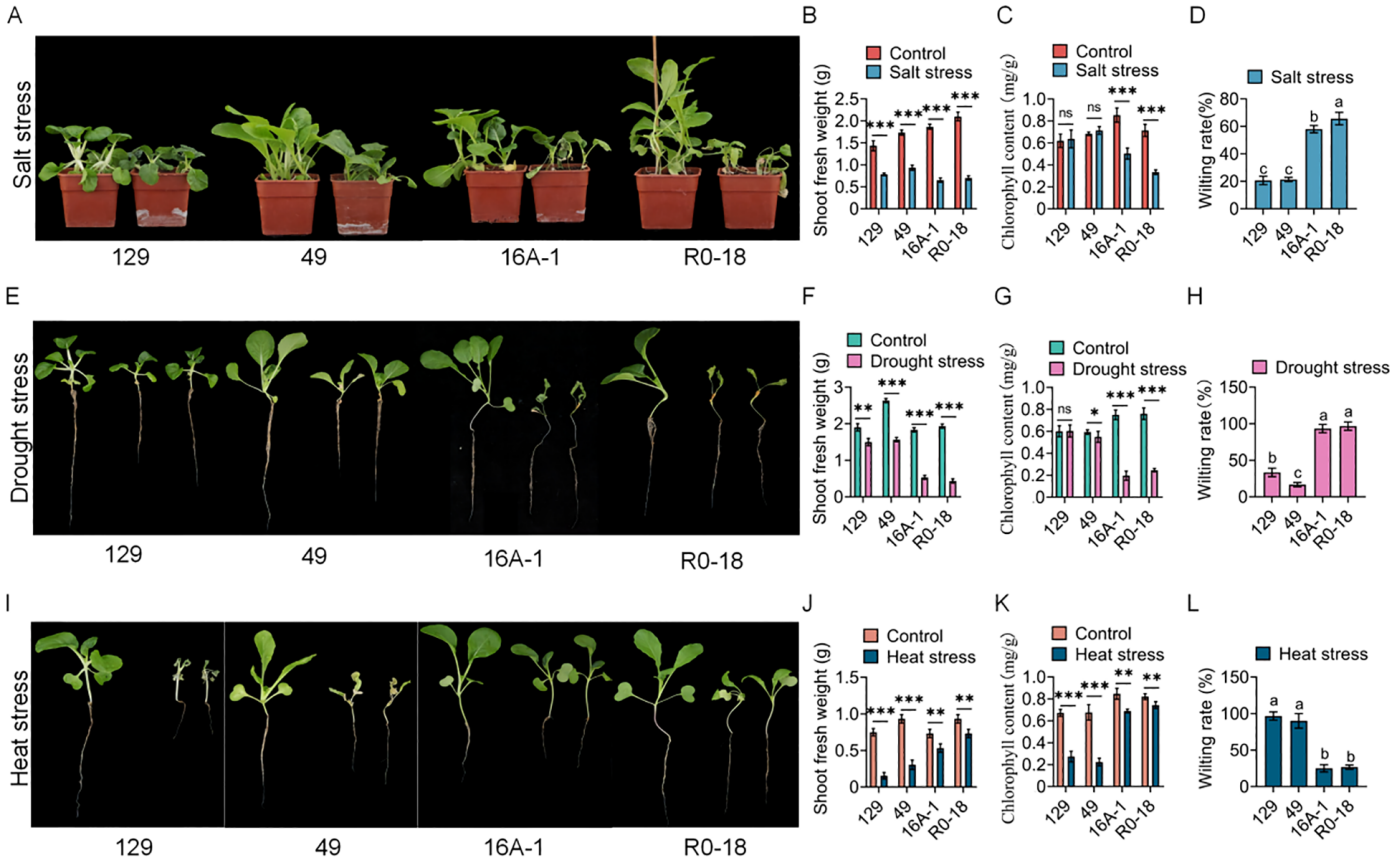
Phenotypic and physiological characterization of four *B. rapa* inbred lines under salt, drought, and heat stresses. **(A)** Phenotypes of seedlings after 7 days of 250 mM NaCl treatment. **(B–D)** Shoot fresh weight **(B)**, chlorophyll content **(C)**, and wilting rate **(D)** under salt stress. **(E)** Phenotypes of seedlings after 7 days of 20% PEG6000 treatment. **(F–H)** Shoot fresh weight **(F)**, chlorophyll content **(G)**, and wilting rate **(H)** under drought stress. **(I)** Phenotypes of seedlings after 12 h of 40°C heat stress followed by 7 days of recovery at 22°C. **(J–L)** Shoot fresh weight **(J)**, chlorophyll content **(K)**, and wilting rate **(L)** under heat stress. Salt/drought tolerant lines (129 and 49) and thermotolerant lines (16A-1 and R0-18) are indicated. Data are presented as mean ± SD (n = 3 biological replicates, each with 10 plants). Different lowercase letters above bars indicate significant differences among genotypes under the same stress condition (one-way ANOVA, Duncan’s multiple range test, *P* < 0.05).

To further investigate this apparent negative association, we selected four representative lines (129, 49, 16A-1, and R0-18) from the two groups and subjected them to salt, drought, and heat stress, respectively. Lines 129 and 49 demonstrated robust salt tolerance under 250 mM NaCl, as evidenced by a low wilting rate (~20%) and stable chlorophyll content (no significant difference vs. control, *P* > 0.05), despite a 50% reduction in aboveground fresh weight (**Figures 1A–D**). Similarly, under drought stress (20% PEG6000), line 129 exhibited minimal inhibition of aboveground biomass, while line 49 displayed the lowest wilting rate (~15%) and maintained chlorophyll levels comparable to controls (**Figures 1E–H**). In stark contrast, both lines showed extreme sensitivity to heat stress (40°C), with nearly complete wilting (95% wilting rate), a 62% reduction in chlorophyll content, and significant declines in aboveground fresh weight after 48 h (**Figures 1I–L**). Conversely, lines 16A-1 and R0–18 exhibited superior thermotolerance, retaining high chlorophyll stability (only 15% degradation) and a low wilting rate (~15%) under heat stress (**Figures 1I–L**). However, these heat-tolerant lines displayed weakened salt/drought adaptability, with pronounced growth inhibition and wilting under salinity and drought conditions (**Figures 1A–H**). These physiological metrics are consistent with a negative association between salt/drought tolerance and thermotolerance in the tested *B. rapa* germplasm.

### 2.2 Dynamic transcriptional reprogramming under salt, drought, and heat stress

#### 2.2.1 Salt stress response

**Figure 2:**
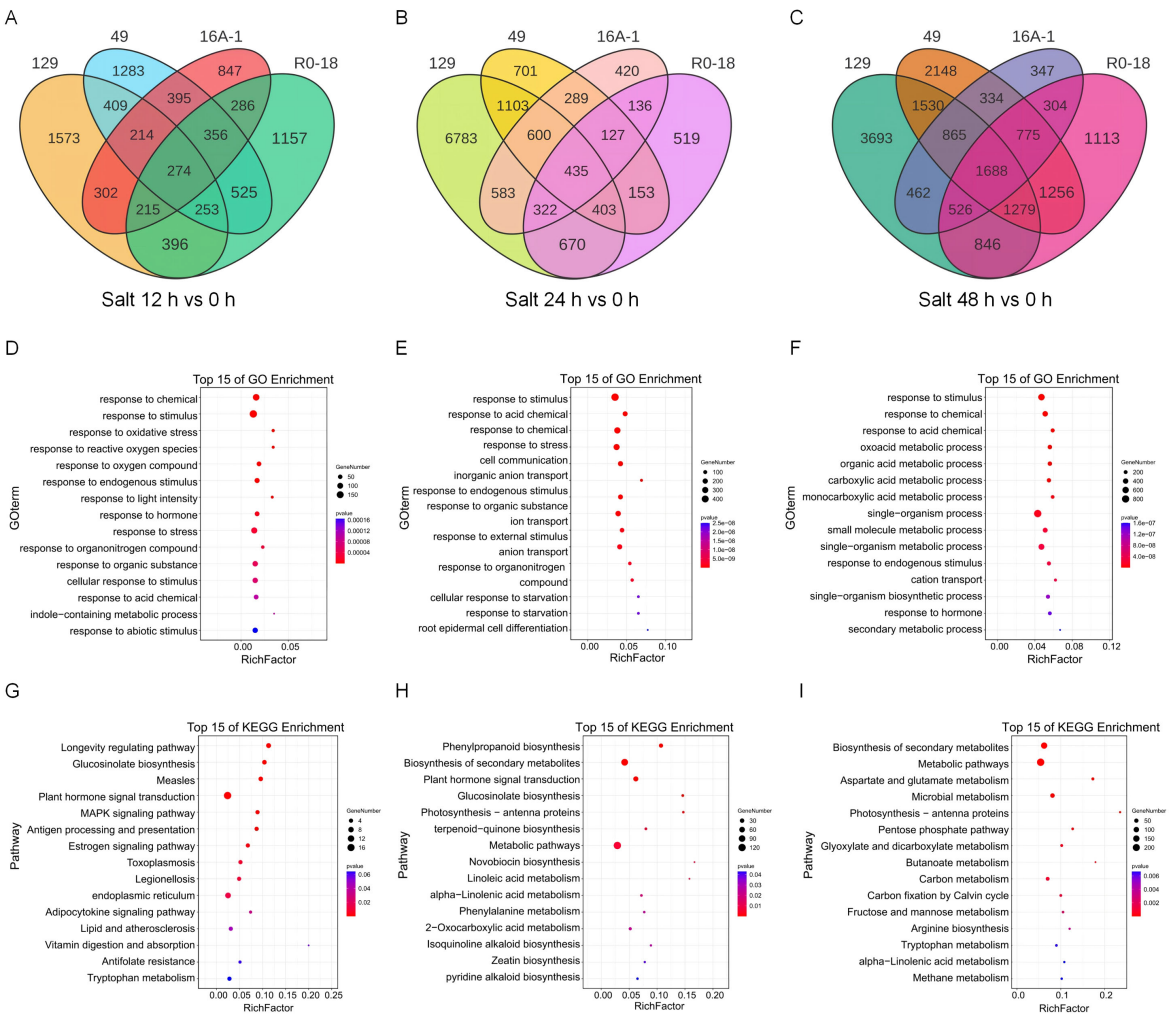
Transcriptomic dynamics of *B. rapa* under salt stress (250 mM NaCl). **(A–C)** Venn diagrams showing the numbers of differentially expressed genes (DEGs) identified at 12 h **(A)**, 24 h **(B)** and 48 h **(C)** versus 0 h (control) in the four genotypes (129, 49, 16A-1 and R0-18). The overlapping regions indicate DEGs shared among genotypes, while non−overlapping regions represent genotype−specific DEGs. **(D–F)** Gene Ontology (GO) enrichment analysis of DEGs specifically upregulated in salt−tolerant lines (129 and 49) at 12 h **(D)**, 24 h **(E)** and 48 h **(F)**. Only the top 15 enriched GO terms are shown (*P* < 0.05). **(G–I)** KEGG pathway enrichment analysis of DEGs specifically upregulated in salt−tolerant lines (129 and 49) at 12 h **(G)**, 24 h **(H)** and 48 h **(I)**. The size of the dots represents the number of genes, and the color gradient indicates the significance level (*P*-value). Pathways related to hormone signaling are highlighted. DEGs were defined as |log_2_FC| ≥ 1 and adjusted *P* < 0.05 (Benjamini−Hochberg correction).

Time-course transcriptome analysis of four *B. rapa* inbred lines under 250 mM NaCl stress revealed temporal changes in gene expression. The number of shared differentially expressed genes (DEGs) across all lines progressively increased from 274 at 12 h to 1,688 at 48 h. Salt-tolerant genotypes (129 and 49) showed greater transcriptional plasticity, with DEGs expanding from 409 to 1,530 over the same period (**Figures 2A–C**; **Supplementary Figure 2**; **Supplementary Table 1**). Phase-specific GO enrichment indicated a temporal cascade: at 12 h, enrichment of ROS scavenger genes (*APX*, *SOD*) suggested early oxidative defense (**Figure 2D**; **Supplementary Figures 3A, B**); at 24 h, genes involved in acid chemical response pathways were overrepresented, pointing to mid-phase cellular homeostasis (**Figure 2E**); and at 48 h, root development regulators (*WOX5*, *LBD16*) were enriched, implying late structural adaptation (**Figure 2F**; **Supplementary Figures 3C, D**). KEGG analysis further identified pathways related to hormone signaling crosstalk (ABA-JA-ethylene) (**Figures 2G–I**; **Supplementary Figure 4**), endoplasmic reticulum stress adaptation (*BiP*, *PDIL*) (**Figure 2G**; **Supplementary Figures 3E, F**), and nitrogen metabolic reprogramming (*GS/GOGAT*) (**Figure 2I**; **Supplementary Figure 3G**). Together, these temporal patterns are consistent with a multi-stage response: early (0–12 h) activation of ABA-mediated and ROS-scavenging pathways, followed by metabolic optimization involving MAPK and nitrogen networks (12–24 h), and later (>24 h) changes in root development-associated transcripts.

#### 2.2.2 Drought stress response

**Figure 3:**
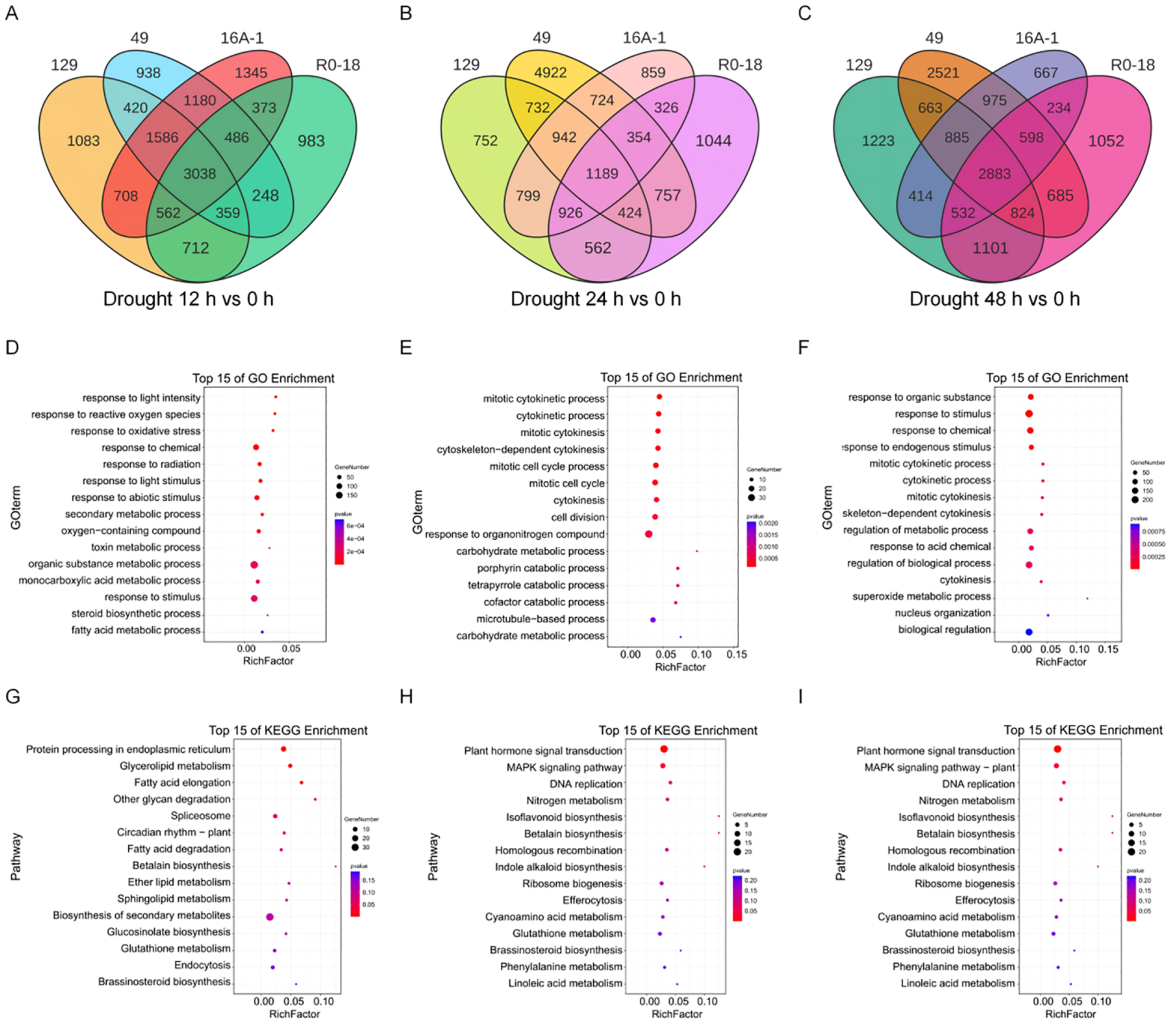
Transcriptomic dynamics of *B. rapa* under drought stress (20% PEG6000). **(A–C)** Venn diagrams showing the numbers of differentially expressed genes (DEGs) identified at 12 h **(A)**, 24 h **(B)** and 48 h **(C)** versus 0 h (control) in the four genotypes (129, 49, 16A-1 and R0-18). The overlapping regions indicate DEGs shared among genotypes, while non-overlapping regions represent genotype-specific DEGs. **(D–F)** Gene Ontology (GO) enrichment analysis of DEGs specifically upregulated in drought tolerant lines (129 and 49) at 12 h **(D)**, 24 h **(E)** and 48 h **(F)**. Only the top 15 enriched GO terms are shown (*P* < 0.05). **(G–I)** KEGG pathway enrichment analysis of DEGs specifically upregulated in drought tolerant lines (129 and 49) at 12 h **(G)**, 24 h **(H)** and 48 h **(I)**. The size of the dots represents the number of genes, and the color gradient indicates the significance level (*P*-value). DEGs were defined as |log_2_FC| ≥ 1 and adjusted *P* < 0.05 (Benjamini-Hochberg correction).

Under drought stress, the common DEGs across the four lines showed a dynamic pattern of ‘rapid response-readjustment-reactivation’, with numbers of 3,038 at 12 h, 1,189 at 24 h, and 2,883 at 48 h (**Figures 3A–C**; **Supplementary Figure 5**; **Supplementary Table 2**). Drought tolerant lines 129/49 showed a time-specific DEG changes (12 h/420→24 h/732→48 h/663) compared with the sensitive lines (**Figures 3A–C**). Functional enrichment analysis showed that at 12 h, drought-tolerant lines activated genes involved in ‘response to reactive oxygen species’, including photoprotection (*HY5*) and ROS scavenging (*APX1*) (**Figure 3D**; **Supplementary Figures 6A, B**). At 24 h, enrichment of mitotic cytokinetic process genes (*CYCB*) suggested cell cycle regulation, possibly reflecting resource allocation (**Figure 3E**; **Supplementary Figure 6C**). At 48 h, ‘response to organic substance’ was enriched, with accumulation of transcripts for osmoregulatory substances (*P5CS*) and secondary metabolites (*DODA*) (**Figure 3F**; **Supplementary Figures 6D, G**). KEGG analysis pointed to pathways including endoplasmic reticulum protein processing (*BIP*), brassinosterol synthesis (*DWF5*), and betalain metabolism (**Figures 3G–I**; **Supplementary Figures 6E, F**, **7**). Notably, a *DODA*-encoding gene, which in other plant families functions in betalain synthesis, was induced under drought; however, since *Brassicaceae* do not produce betalains, the specific role of this gene in *B. rapa* drought response remains unknown and requires further investigation.

#### 2.2.3 Heat stress response

**Figure 4:**
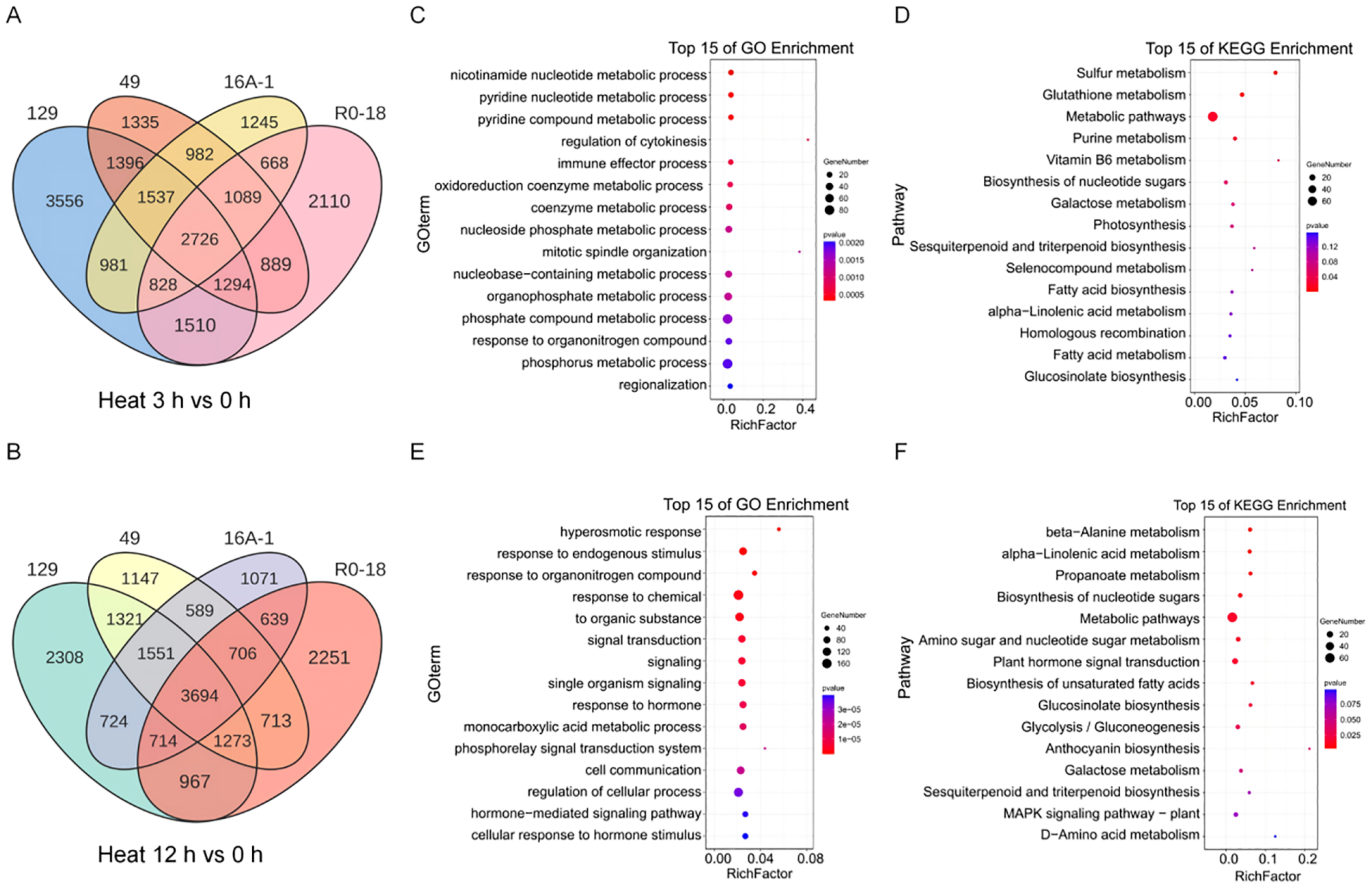
Transcriptional regulation of thermotolerance in *B. rapa* under heat stress (40°C). **(A)** Venn diagram showing the numbers of differentially expressed genes (DEGs) identified at 3 h versus 0 h (control) in the four genotypes (129, 49, 16A-1 and R0-18). The overlapping regions indicate DEGs shared among genotypes, while non-overlapping regions represent genotype-specific DEGs. **(B)** Venn diagram of DEGs identified at 12 h versus 0 h. **(C, D)** Gene Ontology (GO) enrichment analysis **(C)** and KEGG pathway enrichment analysis **(D)** of DEGs specifically upregulated in thermotolerant lines (16A-1 and R0-18) at 3 h. **(E, F)** Gene Ontology (GO) enrichment analysis **(E)** and KEGG pathway enrichment analysis **(F)** of DEGs specifically upregulated in thermotolerant lines (16A-1 and R0-18) at 12 h. Only the top 15 enriched GO terms are shown (*P* < 0.05). The size of the dots represents the number of genes, and the color gradient indicates the significance level (*P*-value). DEGs were defined as |log_2_FC| ≥ 1 and adjusted *P* < 0.05 (Benjamini-Hochberg correction).

Transcriptomic profiling under 40°C heat stress revealed dynamic changes in gene expression. At 3 h post-treatment, 2,726 genes were commonly induced across all four genotypes, while 668 genes were specifically upregulated in thermotolerant lines (16A-1, R0-18) (**Figure 4A**). GO enrichment analysis of these 668 genes showed significant associations with “nicotinamide nucleotide metabolic process” and “pyridine nucleotide metabolic process” (**Figure 4C**). KEGG pathway analysis identified enrichment in “sulfur metabolism” and “glutathione metabolism” (**Figure 4D**). The early induced genes included *heat shock factors* (*HSFs*), *WRKY*, and *NAC* family members, suggesting a conserved early transcriptional response (**Supplementary Figures 8**, **9A–E**; **Supplementary Table 3**). By 12 h, the total number of heat-induced genes increased to 3, 694, with thermotolerant genotypes exhibiting 639 unique DEGs (**Figure 4B**). Functional enrichment analysis of these late-phase DEGs revealed associations with ‘hyperosmotic response’ and, ‘response to endogenous stimulus’, while KEGG analysis implicated ‘beta-Alanine metabolism’ and the ‘alpha-Linolenic acid metabolism’ (**Figures 4E, F**). Notably, several genes, including *PMEI7*, *PHOS34*, *GGT3* and *JAR1*, showed significant expression differences, with markedly higher upregulation in thermotolerant lines than in heat-sensitive lines (**Supplementary Figures 9F–I**).

### 2.3 Hormonal crosstalk underlies stress-specific responses

**Figure 5:**
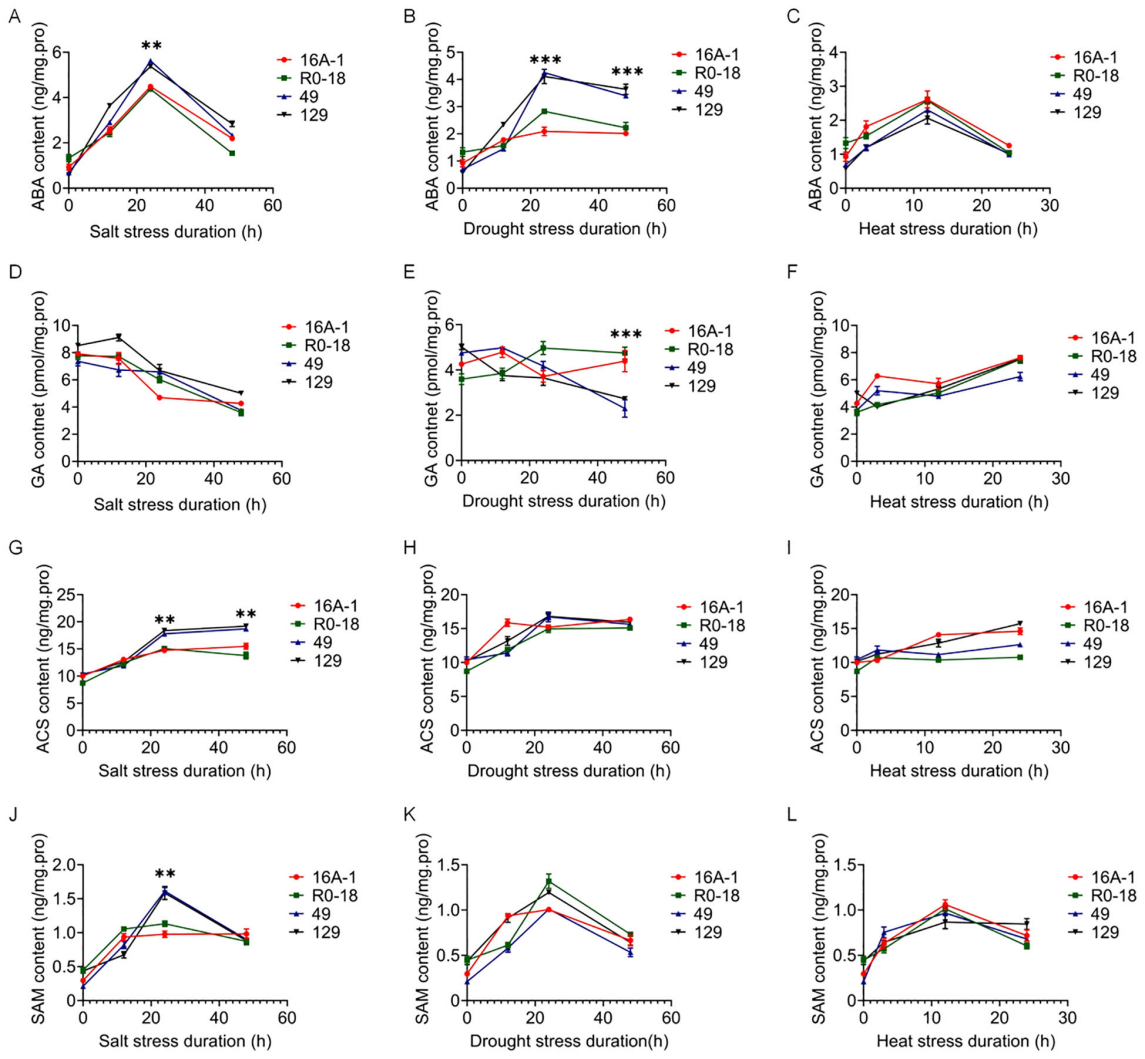
Hormonal dynamics of four *B. rapa* inbred lines under salt, drought and heat stress. **(A–C)** ABA content under salt **(A)**, drought **(B)** and heat stress **(C)**. **(D–F)** GA content under salt **(D)**, drought **(E)** and heat stress **(F)**. **(G–I)** ACS content under salt **(G)**, drought **(H)** and heat stress **(I)**. **(J–L)** SAM content under salt **(J)**, drought **(K)** and heat stress **(L)**. Salt/drought tolerant lines (129 and 49) and thermotolerant lines (16A-1 and R0-18) are indicated by different line colors. Data are presented as mean ± SD (n = 3 biological replicates, each with 10 plants). Asterisks indicate significant differences between tolerant and sensitive lines at the same time point (**P < 0.01, ***P < 0.001; one-way ANOVA, Duncan’s multiple range test). SAM, S-adenosylmethionine; ACS, 1-aminocyclopropane-1-carboxylic acid synthase.

KEGG pathway enrichment analysis indicated that plant hormone signal transduction pathways were involved in the responses to salt, drought and heat stress in *B. rapa*. Time-course profiling of hormone dynamics revealed that cytokinin (CTK), jasmonic acid (JA) and brassinosteroid (BR) showed notable changes under all three stress conditions, whereas other hormones exhibited more stress specific patterns (**Table 1**, **Supplementary Figures 4**, **7**, **10**). To validate the hormone-related signaling pathways identified by transcriptome analysis, we measured ABA, GA, and the ethylene biosynthesis-related metabolites (SAM and ACS) in four genotypes (129, 49, 16A-1, R0-18) under salt, drought and heat stress (**Figure 5**). ABA content initially increased and then decreased under all three stresses. However, significant genotypic differences were observed only under salt and drought stress. Under salt stress at 24 h, ABA levels of 129 and 49 were significantly higher than in 16A-1 and R0-18 (**Figure 5A**). Under drought stress, ABA levels in 129 and 49 remained higher at both 24 h and 48 h, whereas no genotypic difference was detected under heat stress (**Figures 5B, C**). GA content decreased under salt stress, but the reduction did not differ significantly among genotypes (**Figure 5D**). Under heat stress, GA levels remained comparable to the control (**Figure 5F**). Notably, under drought stress, GA content was significantly down-regulated in lines 129 and 49 but remained stable in 16A-1 and R0-18 (**Figure 5E**). For ethylene, we measured the content of its biosynthetic precursor S-adenosylmethionine (SAM) and the activity of the key enzyme 1-aminocyclopropane-1-carboxylic acid synthase (ACS). Under salt stress, both SAM and ACS accumulated in a time-dependent manner in lines 129 and 49, reaching a peak at 24 h, with levels significantly higher than those in 16A-1 and R0-18 (**Figures 5G, J**). This temporal pattern was similar to that of ABA. Under drought and heat stress, no significant differences in SAM or ACS levels were observed among the four lines (**Figures 5H, I, K, L**).

**Table 1:** KEGG pathway enrichment of plant hormone signal transduction under salt, drought and heat stress at different time points.

Stress type	Time point	Enriched hormone signaling pathways
Salt stress	12 h vs 0 h	JA
	24 h vs 0 h	CTK, ABA, JA, ETH, BR
	48 h vs 0 h	CTK, ABA, JA, ETH, BR
Drought stress	12 h vs 0 h	CTK, BR
	24 h vs 0 h	CTK, BR
	48 h vs 0 h	CTK, GA, ABA, BR, JA, SA
Heat stress	12 h vs 0 h	CTK, BR, JA, SA

Hormone related pathways enriched in DEGs specifically upregulated in salt tolerant lines (129 and 49) under 250 mM NaCl (12, 24 and 48 h vs 0 h), drought tolerant lines (129 and 49) under 20% PEG6000 (12, 24 and 48 h vs 0 h), and thermotolerant lines (16A-1 and R0-18) under 40°C heat stress (12 h vs 0 h) are summarized. Only pathways with *P* < 0.05 (Benjamini-Hochberg corrected) are shown. CTK, cytokinin; JA, jasmonic acid; ABA, abscisic acid; ETH, ethylene; BR, brassinosteroid; GA, gibberellin; SA, salicylic acid.

### 2.4 Anthocyanin biosynthesis is associated with stress responses via *BraA10g024990.3C* (*CHS*)

**Figure 6:**
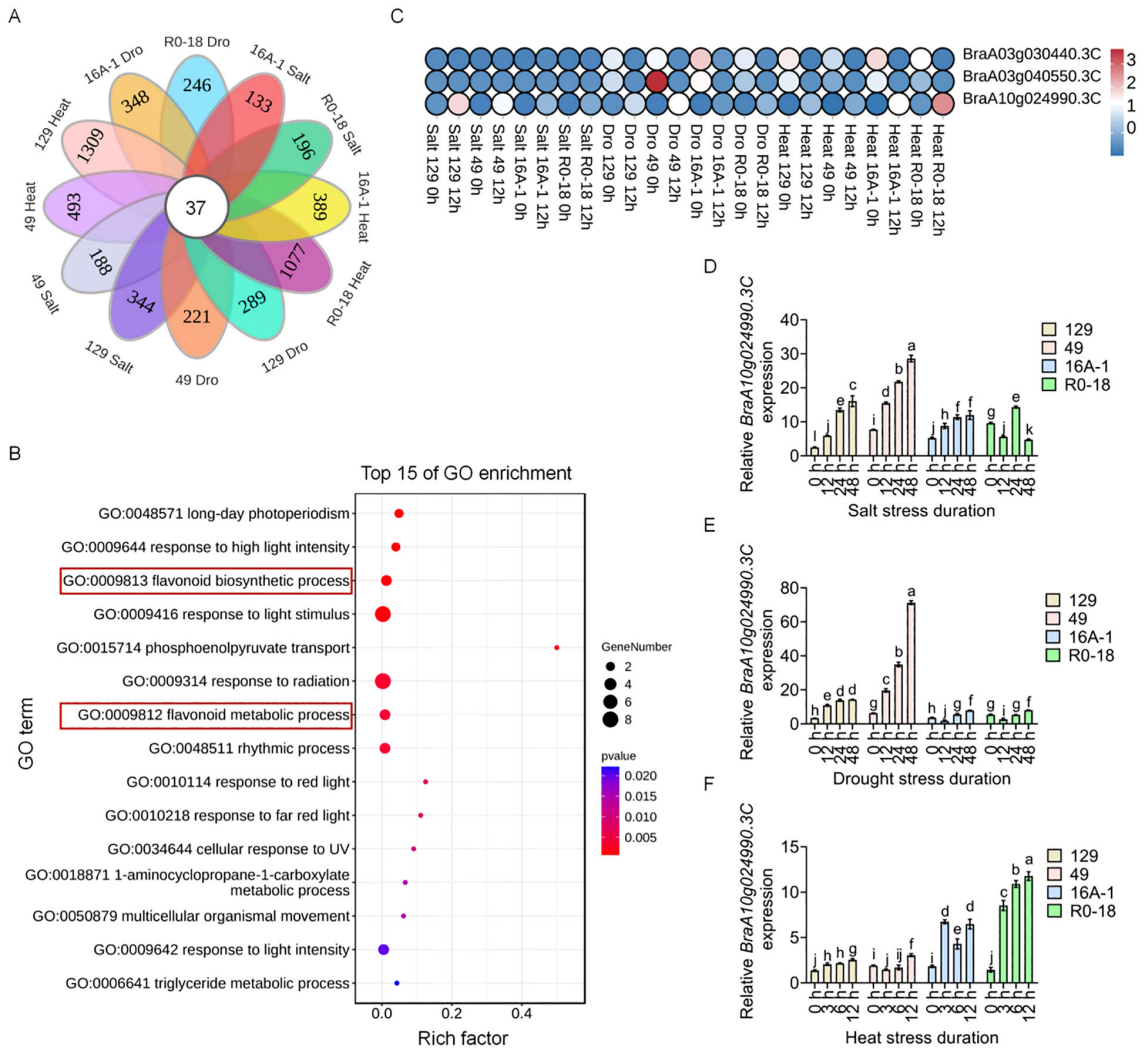
Integrated functional characterization of common DEGs and temporal expression of flavonoid biosynthesis genes under salt, drought and heat stress. **(A)** Venn diagram showing common DEGs identified across salt, drought and heat stress at 12 h versus 0 h. The overlapping region indicates 37 genes that were consistently differentially expressed across all three stress conditions. **(B)** GO enrichment analysis of the 37 common DEGs. Only the top 15 enriched GO terms are shown (*P* < 0.05). **(C)** Heatmap of expression profiles of flavonoid biosynthesis-related genes among the 37 common DEGs in the four genotypes (129, 49, 16A-1 and R0-18) under salt, drought and heat stress. Red and blue indicate high and low expression levels, respectively. **(D–F)** Temporal expression patterns of *BraA10g024990.3C* (CHS) under salt **(D)**, drought **(E)** and heat stress **(F)**. Data are presented as mean ± SD (n = 3 biological replicates, each with 10 plants). Different lowercase letters indicate significant differences among genotypes at the same time point (one-way ANOVA, Duncan’s multiple range test, *P* < 0.05). DEGs were defined as |log_2_FC| ≥ 1 and adjusted *P* < 0.05 (Benjamini-Hochberg correction).

To investigate the molecular responses to salt, drought, and heat stress across four genotypes (16A-1, R0-18, 49, and 129), we performed DEG analysis comparing 12 h stress-treated samples with untreated controls (0 h). A total of 37 genes were consistently differentially expressed across all four genotypes under the three stress conditions (**Figure 6A**). Gene Ontology (GO) enrichment analysis revealed that these genes were primarily associated with ‘long-day photoperiodism’, ‘response to high light intensity’ and ‘flavonoid biosynthetic process’ (**Figure 6B**). Given the well-documented role of flavonoids in plant stress adaptation, we focused on the three genes annotated to the ‘flavonoid biosynthetic process’ pathway. Among these genes, *BraA10g024990.3C* exhibited distinct expression profiles under different stresses (**Figure 6C**). Under salt and drought stress at 12 h, *BraA10g024990.3C* showed significant upregulation in stress-tolerant genotypes 129 and 49 (2.1- to 5.5-fold vs. control, *P* < 0.01), whereas heat stress induced pronounced upregulation in thermotolerant genotypes 16A-1 and R0-18 (3.5- to 7.8-fold, *P* < 0.001) (**Figures 6D–F**). Temporal expression analysis further demonstrated that *BraA10g024990.3C* was predominantly upregulated during salt and drought stress, with stronger induction in 129 and 49 (peak 10.3-fold at 48 h, *P* < 0.001) (**Figures 6D–F**). Conversely, under heat stress, its expression was markedly elevated in 16A-1 and R0-18 (7.8-fold at 12 h, *P* < 0.001) (**Figures 6D–F**). These expression patterns indicate that *BraA10g024990.3C* responds to multiple stresses in a genotype- and stress-dependent manner.

Bioinformatic analysis identified *BraA10g024990.3C* encodes chalcone synthase (CHS), a key enzyme catalyzing anthocyanin biosynthesis in leaves and stems. The protein comprises 395 amino acids, featuring conserved Chalcone and stilbene synthases (CHS/STS) domains at both N- and C-termini (**Supplementary Figure 11**). Amino acid sequence alignment analysis revealed high homology (88.55% sequence similarity) with *Arabidopsis thaliana* CHS (AT5G13930), suggesting functional conservation (**Supplementary Figure 12**). Through cis-regulatory element prediction analysis of the *CHS* promoter region (–2000 bp relative to TSS), we identified both phytohormone-responsive motifs (ABRE, ERE, MYB/MYC-binding sites, and TCA-element) and abiotic stress-responsive cis-elements (MBS, STRE, and LTR) (**Supplementary Figure 13**). The cis-element profiling is consistent with a potential role of *CHS* in stress adaptation in *B. rapa.*

**Figure 7:**
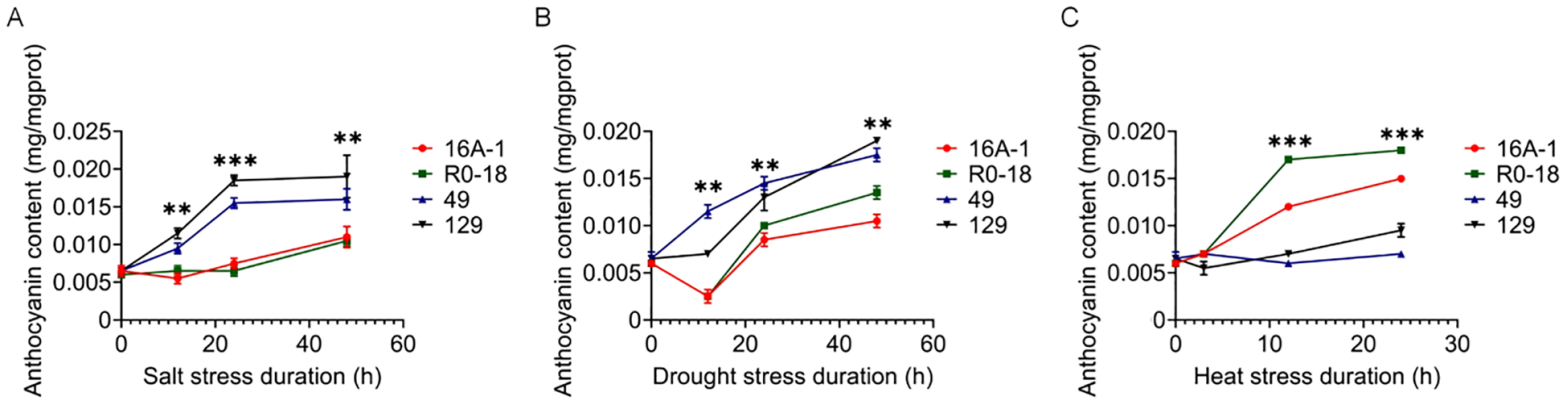
Anthocyanin accumulation in *B. rapa* under salt, drought and heat stress. **(A)** Anthocyanin content under salt stress (250 mM NaCl) at 12, 24 and 48 h post-treatment. **(B)** Anthocyanin content under drought stress (20% PEG6000) at 12, 24 and 48 h post-treatment. **(C)** Anthocyanin content under heat stress (40°C) at 3 and 12 h post-treatment. Salt/drought-tolerant lines (129 and 49) and thermotolerant lines (16A-1 and R0-18) are indicated by different line colors. Data are presented as mean ± SD (n = 3 biological replicates, each with 10 plants). Asterisks indicate significant differences between tolerant and sensitive lines at the same time point (**P < 0.01, ***P < 0.001; one-way ANOVA, Duncan’s multiple range test).

Quantification of anthocyanin levels under stress treatments showed that salt- and drought-tolerant genotypes 129 and 49 exhibited about 1.4- and 1.6-fold higher anthocyanin accumulation compared to 16A-1 and R0–18 after 48 h treatment (*P* < 0.01) (**Figures 7A, B**). Conversely, thermotolerant genotypes 16A-1 and R0–18 showed 2.1- to 2.8-fold elevated anthocyanin levels under heat stress relative to 129 and 49 after 12 h heat stress (*P* < 0.001) (**Figure 7C**). These results indicate that anthocyanin accumulation is positively correlated with stress resilience in the tested genotypes under the respective stress conditions.

### 2.5 Differential responsiveness of the *CHS*-anthocyanin pathway to exogenous hormones

**Figure 8:**
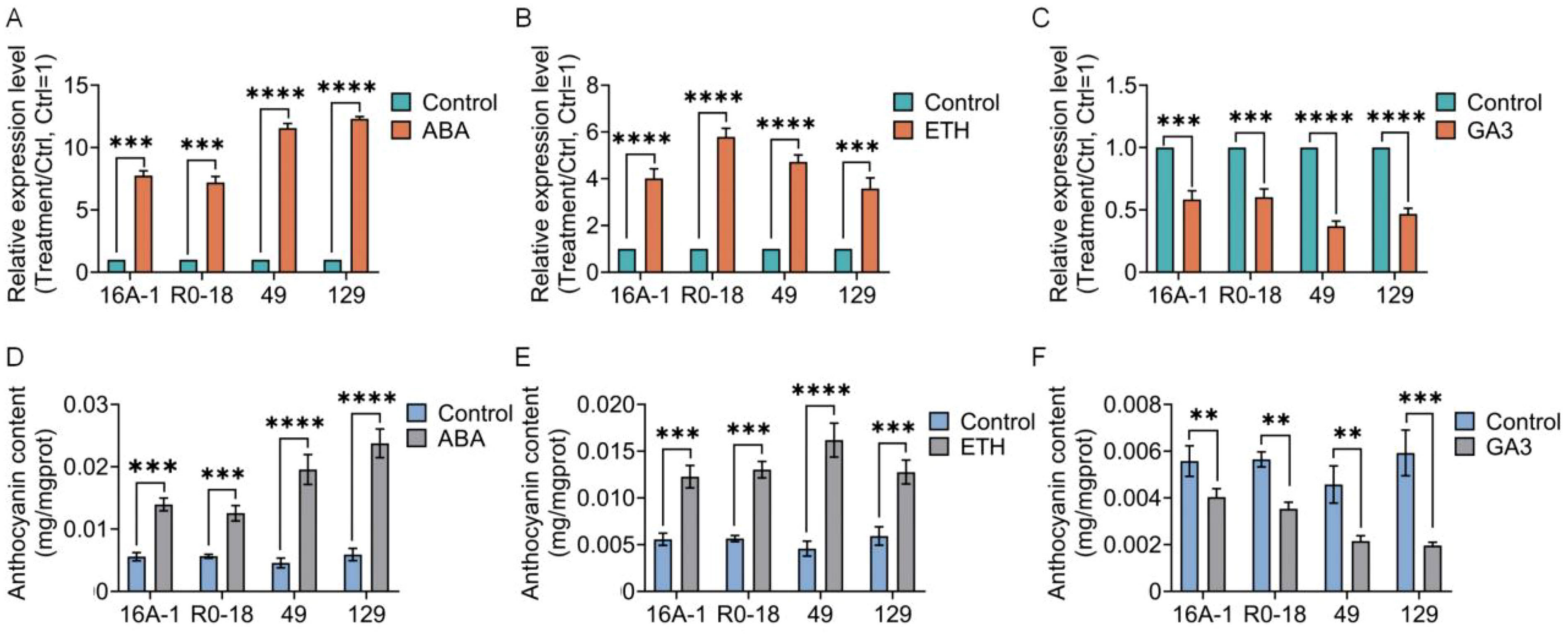
Hormonal regulation of *CHS* expression and anthocyanin biosynthesis. **(A–C)** Relative expression of *BraA10g024990.3C (CHS)* under exogenous ABA **(A)**, ethylene (ethylene-releasing agent, **(B)**), and GA3 **(C)** treatments. **(D–F)** Anthocyanin content under ABA **(D)**, ethylene **(E)**, and GA3 **(F)** treatments. Four genotypes (129, 49, 16A-1 and R0-18) were treated with 100 µM ABA, 200 µM ethephon, or 100 µM GA3, with distilled water as the control. Samples were collected at 12 h post-treatment. Data are presented as mean ± SD (n = 3 biological replicates, each with 10 plants). Asterisks indicate significant differences compared to the corresponding control (**P < 0.01, ***P < 0.001, ****P < 0.0001; Student’s t-test). *CHS* expression was normalized to the internal reference gene *BraActin*. Anthocyanin content was measured as absorbance at 530 nm and normalized to fresh weight (FW).

Consistent with established phytohormonal regulation of flavonoid biosynthesis, exogenous hormone treatments were applied to four *B. rapa* inbred lines to examine their effects on *BraA10g024990.3C* (*CHS*) expression and anthocyanin accumulation. ABA pretreatment upregulated *CHS* expression and anthocyanin content across all lines (*P* < 0.01). The induction was significantly higher in salt/drought-tolerant lines 129/49 than in thermotolerant lines 16A-1/R0-18 (*CHS*: 11.6 – 12.3-fold vs. 7.21 – 7.76-fold; anthocyanins: 4.01 – 4.28-fold vs. 2.20 – 2.51-fold (**Figures 8A, D**). Ethephon (an ethylene-releasing agent) also enhanced *CHS* and anthocyanins in all inbred lines (*CHS*:3.59 – 5.79-fold; anthocyanins: 2.15 – 3.54-fold), with no significant genotypic difference observed (**Figures 8B, E**). In contrast, GA treatment suppressed both parameters (*CHS*: 0.37 – 0.60-fold; anthocyanins: 0.33 – 0.72-fold) (**Figures 8C, F**).

In summary, exogenous ABA and ethylene (ethephon) both promoted *CHS* expression and anthocyanin accumulation, whereas GA suppressed these parameters. The magnitude of ABA-induced *CHS* upregulation and anthocyanin increase was significantly greater in the salt/drought−tolerant lines (129 and 49) than in the thermotolerant lines (16A−1 and R0−18), whereas ethylene induced comparable responses across all four genotypes. These results indicate that the responsiveness of the *CHS*−anthocyanin pathway to specific hormones differs between genotypic groups and stress adaptation types.

## 3 Discussion

### 3.1 An apparent negative association between salt/drought tolerance and thermotolerance in *B. rapa*

This study provides evidence for an apparent negative association between salt/drought tolerance and thermotolerance across diverse *B. rapa* inbred lines. Physiological validation using four representative lines (salt/drought-tolerant: 129, 49; thermotolerant: 16A-1, R0-18) supported this observation (**Figure 1**). Salt/drought-tolerant lines maintained low wilting rates, stable chlorophyll content, and minimized biomass loss under high salinity (250 mM NaCl) or osmotic stress (20% PEG6000), yet exhibited extreme susceptibility to heat stress (40°C), manifesting as near-complete wilting and severe chlorophyll degradation (62% reduction) (**Figure 1**). Conversely, thermotolerant lines displayed robust chlorophyll stability (only 15% degradation) and low wilting under heat stress but suffered significant growth inhibition under salt/drought conditions (**Figure 1**). Correlation analysis across 11 lines further supported this negative association, with Pearson correlation coefficients of r = –0.555 between salt and heat stress responses and r = –0.339 between drought and heat stress responses (**Supplementary Figure 1D**). This physiological divergence may reflect different resource allocation priorities: salt/drought-adapted genotypes invest in osmotic adjustment and reactive oxygen species (ROS) detoxification, whereas thermotolerant genotypes prioritize maintaining protein conformational stability and membrane integrity. Such inherently different strategies may create a potential conflict when plants face contrasting stresses sequentially or simultaneously.

### 3.2 Dynamic transcriptional reprogramming and hormonal patterns associated with stress-specific adaptation

#### 3.2.1 Triphasic transcriptional patterns in salt stress adaptation

Salt stress elicited a time-dependent increase in DEGs in tolerant lines (129, 49). The temporal patterns were consistent with a multi-stage response: at 12 h, activation of ROS scavengers (e.g., *APX*, *SOD*) was observed (**Figure 2D**; **Supplementary Figures 3A, B**); at 24 h, enrichment of cellular homeostasis pathways occurred; and at 48 h, changes in root development regulators (e.g., *WOX5*, *LBD16*) were evident (**Figure 2F**; **Supplementary Figures 3C, D**). KEGG analysis identified pathways related to hormone signaling crosstalk (ABA-JA-ethylene), endoplasmic reticulum stress adaptation (*BiP*, *PDIL*) (**Figure 2G**; **Supplementary Figures 3E, F**), and nitrogen metabolic reprogramming (*GS/GOGAT*) (**Figure 2I**; **Supplementary Figure 3G**). While aspects of this response are conserved (e.g., ABA signaling (Lamers et al., 2025), ER stress (Liu et al., 2007)), the coordinated changes in nitrogen metabolism and root developmental-associated transcripts in *B. rapa* may represent a distinct strategy for long-term salinity resilience.

#### 3.2.2 Temporal modules associated with drought stress response

The drought response exhibited a distinct ‘rapid response - readjustment - reactivation’ DEG pattern. Tolerant lines (129/49) showed stage-specific changes: (i) at 12 h, enrichment of photoprotection (*HY5*) and ROS clearance (*APX1*) genes (**Figure 3D**; **Supplementary Figures 6A, B**); (ii) at 24 h, cell cycle regulation (*CYCB*)-associated transcripts (**Figure 3E**; **Supplementary Figure 6C**); and (iii) at 48 h, accumulation of transcripts for osmolytes (*P5CS*) and secondary metabolites genes (**Figure 3F**; **Supplementary Figure 6D**). KEGG analysis highlighted pathways including ER protein processing (*BiP*), brassinosteroid synthesis (*DWF5*), and betalain metabolism (**Figures 3G–I**; **Supplementary Figures 6E, F**). Notably, a *DODA*-encoding gene, which in other plant families functions in betalain synthesis, was induced under drought in the tolerant lines (**Supplementary Figure 6G**). However, since *Brassicaceae* do not produce betalains (Timoneda et al., 2019), the specific function of this *DODA*-homolog in *B. rapa* remains unknown and warrants further investigation.

#### 3.2.3 Temporal patterns in heat stress response

Heat stress triggered a conserved early response across all genotypes at 3 h, characterized by rapid induction of approximately 2, 726 genes, predominantly transcription factors (*HSFs* (Guo et al., 2016)*, WRKY* (Li et al., 2010)*, NAC* (Ren et al., 2021)) (**Figure 4A**; **Supplementary Figures 8**, **9A–E**). By 12 h, thermotolerant lines (16A-1, R0-18) exhibited 639 unique DEGs, enriched in hormone signal transduction (**Figures 4E, F**, **Supplementary Figures 9F–I**). This temporal shift suggests that sustained thermotolerance may involve genotype-specific metabolic reprogramming beyond the initial HSP-dominated shock response commonly observed in models like *Arabidopsis* (Yamada et al., 2007).

#### 3.2.4 Hormonal signatures associated with stress specificity

Integrated hormone profiling revealed distinct spatio-temporal patterns. ABA levels were significantly higher in salt/drought-tolerant lines under salt and drought stress. CTK and BR showed dynamic changes across all three stresses. Under salt stress, the ethylene biosynthesis-related metabolites SAM and ACS activity increased predominantly in tolerant lines. Under drought stress, GA levels decreased. These observations suggest that different hormones or their biosynthesis pathways are associated with specific stress conditions. However, given the correlative nature of these data (e.g., ethylene gas was not directly measured), the proposed regulatory relationships require further experimental validation.

### 3.3 Chalcone synthase as a candidate gene associated with stress responses via anthocyanin metabolism

#### 3.3.1 Genotype- and stress-specific expression of *BraA10g024990.3C* (*CHS*)

The gene *BraA10g024990.3C*, encoding chalcone synthase (*CHS*), a key anthocyanin biosynthetic enzyme, showed significant genotype-specific upregulation: under salt/drought stress in tolerant lines (129, 49) and under heat stress in thermotolerant lines (16A-1, R0-18). *CHS* expression correlated with the observed stress-specific anthocyanin accumulation patterns (Sun and Liang, 2019). Its high sequence similarity (88.55%) and conserved CHS/STS domain with *Arabidopsis* CHS (AT5G13930) suggest functional conservation. This genotype-and stress-specific expression profile identifies *CHS* as a candidate regulator that may direct metabolic flux toward protective anthocyanin synthesis in a context-dependent manner. However, direct functional evidence is needed to confirm its causal role.

#### 3.3.2 A hormone-anthocyanin axis potentially associated with the negative association

Exogenous hormone treatments and endogenous profiling revealed a regulatory pattern for *CHS* and anthocyanins: ABA and ethylene (or its biosynthetic precursors) act as inducers, while GA functions as a repressor. The induction by ABA/ethylene was greater in inbred lines naturally tolerant to the corresponding stress (ABA/ethylene in salt/drought-tolerant lines; ethylene in thermotolerant lines under heat). Under salt/drought stress, tolerant lines exhibited elevated ABA coupled with GA depletion, correlating with increased *CHS* expression and anthocyanin mediated antioxidant/osmotic protection. Under heat stress, thermotolerant lines showed ethylene biosynthetic precursors accumulation, which was associated with *CHS* induction and anthocyanin accumulation, potentially contributing to photoprotection and membrane stabilization. These correlative findings suggest that hormone directed reprogramming of anthocyanin metabolism may contribute to a metabolic partition, channeling resources either toward osmotic/oxidative defense (under salt/drought via ABA) or toward thermoprotection (under heat via ethylene), consistent with the observed negative association between salt/drought tolerance and thermotolerance. Future studies employing genetic approaches, such as CRISPR/Cas9-mediated knockout or overexpression of *BraA10g024990.3C* in *B. rapa*, are essential to establish its causal role in mediating this negative association.

## 4 Conclusion

**Figure 9:**
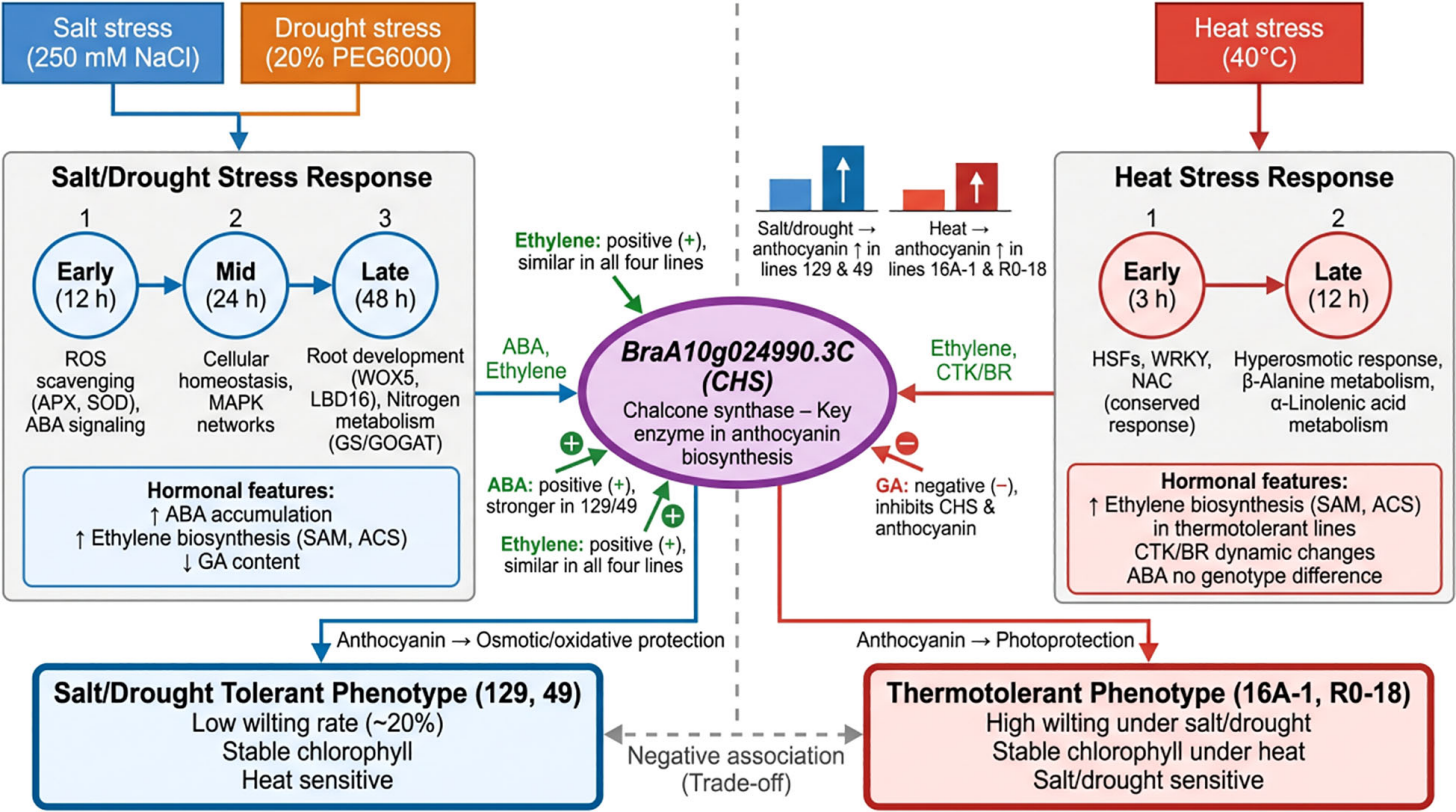
A schematic model of the regulatory network underlying the negative association between salt/drought tolerance and thermotolerance in *B. rapa*. This model integrates key findings from physiological, transcriptomic, hormonal and metabolic analyses of four representative lines (salt/drought-tolerant: 129, 49; thermotolerant: 16A-1, R0-18). Salt and drought stresses trigger ABA accumulation and ethylene biosynthesis, which promote *CHS* expression and anthocyanin accumulation, contributing to osmotic and oxidative protection. Heat stress induces ethylene-related signals and distinct transcriptional programs (*HSFs, WRKY, NAC*), driving *CHS* upregulation and anthocyanin accumulation in thermotolerant lines, potentially contributing to photoprotection. GA acts as a repressor of the *CHS*-anthocyanin pathway. The differential activation of this pathway under contrasting stresses is associated with the observed negative association between salt/drought tolerance and thermotolerance. Solid arrows indicate experimentally supported relationships; dashed arrows represent inferred connections. ABA, abscisic acid; ETH, ethylene; GA, gibberellin; CHS, chalcone synthase; ROS, reactive oxygen species.

Based on the integrated physiological, transcriptomic, hormonal and metabolic analyses, we propose a tentative working model. In this model, divergent hormonal signatures—ABA and ethylene dominance under salt/drought stress versus CTK/BR prominence and ethylene induction under heat stress—are associated with stress specific transcriptional reprogramming (**Figure 9**). This pattern appears to converge on the differential regulation of the *CHS* dependent anthocyanin biosynthesis pathway, which may contribute to partitioning metabolic resources between osmotic/oxidative defense and thermotolerance. Such a resource allocation pattern is consistent with the observed negative association between salt/drought tolerance and thermotolerance in the tested *B. rapa* germplasm.

The hormone-transcriptome-metabolite correlations identified in this study provide a candidate regulatory framework for understanding this negative association. While the correlative nature of these data precludes definitive causal conclusions, the framework highlights several promising targets for future functional validation—including the *CHS* gene, key hormonal nodes (ABA, ethylene and GA signaling components), and stage-specific response modules. Genetic approaches such as CRISPR/Cas9 mediated knockout or overexpression, combined with metabolomic profiling, will be essential to establish causal relationships and to evaluate the potential of these targets for designing next generation *Brassica* crops with enhanced resilience to multiple abiotic stresses.

## 5 Materials and methods

### 5.1 Plant materials and growth conditions

Eleven *B.rapa* inbred lines were maintained at the Beijing Academy of Agriculture and Forestry Sciences. Based on preliminary screening (**Supplementary Figures 1A–C**), four representative genotypes were selected for detailed analyses: salt/drought tolerant lines (129, 49) and thermotolerant lines (16A-1, R0-18). Seeds were surface-sterilized, germinated on moist filter paper at 28°C for 48 h, and transplanted into pots containing a 1:1 mixture of peat and perlite. Plants were grown in a growth chamber at 22 ± 2°C with a 16 h light/8 h dark cycle. Seedlings at the four-leaf stage (25-day-old seedlings) were used for stress treatments.

### 5.2 Stress treatments and sampling

Seedlings of the four selected genotypes at the four-leaf stage were subjected to salt, drought and heat stress treatments. For salt stress, plants were irrigated with 250 mM NaCl solution every three days. This concentration was selected as it has been widely used for salt stress treatment in *B. rapa* transcriptomic studies (Lee et al., 2008). For drought stress, plants were watered once with 20% PEG6000 solution (applied to saturation, with no subsequent re-watering), following the protocol established for *Brassica* species (Ta et al., 2026). For heat stress, seedlings were directly transferred to a growth chamber pre-set at 40°C, a temperature commonly employed for heat stress treatment in *B. rapa* and related species (Zhang et al., 2022).

For salt and drought stress, leaf samples were collected at 0, 12, 24 and 48 h post-treatment for molecular and hormonal analyses. For phenotypic observation (wilting rate, chlorophyll content and shoot fresh weight), a separate batch of plants was treated identically and measured after 7 days of continuous stress. For heat stress, leaves were harvested at 0, 3 and 12 h for molecular analyses; a separate batch of plants was exposed to 40°C for 12 h and then returned to 22°C for 7 days of recovery before phenotypic measurements were taken. The different sampling intervals reflect the distinct temporal dynamics of each stress: salt and drought responses develop relatively slowly (over days), whereas heat stress triggers rapid transcriptional changes within hours, followed by recovery-associated phenotypic changes.

Control groups were treated with distilled water (for salt and drought stress) or maintained at 22°C (for heat stress). All treatments were conducted in a growth chamber with a 16 h light/8 h dark cycle at 22 ± 2°C. For each treatment and each time point, three independent biological replicates were established, each consisting of 10 plants grown in separate pots. Samples for molecular and hormonal analyses were immediately frozen in liquid nitrogen and stored at –80°C until use. Samples for phenotypic measurements were processed fresh immediately after harvest.

### 5.3 Correlation and clustering analysis

Pearson correlation analysis was performed using wilting rates of the 11 *B. rapa* inbred lines under salt, drought and heat stress (Pearson, 1895). Stress tolerance scores for each line were calculated as 1 − wilting rate for each stress condition. To quantify the relationships among stress responses, Pearson correlation coefficients (r) were calculated for all pairwise combinations of salt, drought and heat stress responses. In addition, to evaluate the associations among salt, drought and heat tolerance across the 11 lines, Pearson correlation analysis was performed using the integrated tolerance scores. Based on the resulting correlation patterns, the 11 lines were classified into two distinct groups according to their stress tolerance profiles.

### 5.4 Physiological index measurements

#### 5.4.1 Shoot fresh weight

Shoots were harvested, rinsed, blotted dry, and weighed. Three replicates, each replicate containing 10 seedlings.

#### 5.4.2 Chlorophyll content

Leaf discs (0.2 g) were extracted with 80% acetone, and absorbance was measured at 645 nm and 663 nm using a spectrophotometer (UV-2600, Shimadzu) (Arnon, 1949). Chlorophyll content was calculated as:


$$\text{Chlorophyll}\left(\text{mg}/\text{g}\right)=\frac{\left(20.2\times \text{A}645+8.02\times \text{A}663\right)\times \text{extraction volume }\left(\text{mL}\right)}{\text{sample weight}\left(\text{g}\right)}$$

#### 5.4.3 Wilting rate quantification

The wilting rate of each individual leaf and the total number of leaves per seedling were separately assessed, followed by statistical analysis to determine the overall wilting rate for each plant. Leaf wilting severity was scored using a 5-tier scale:

0: absence of symptoms;
0.25: slight curling or yellowing at the leaf margins only;
0.5: localized leaf deformation or yellow wilting;
0.75: significant shrinkage and deformity of entire leaves or widespread yellow wilting;
1: complete yellowing and death of the whole leaf. The whole-plant wilting rate was calculated as:

$$\text{Wilting Rate}=\frac{\sum \left(\text{Individual leaf scores}\right)}{\text{Total leaf number}}$$

#### 5.4.4 Anthocyanin quantification

Fresh plant tissues were harvested at time points determined according to the distinct temporal dynamics of each stress: 12 h, 24 h, and 48 h for salt and drought stress; 3 h and 12 h for heat stress. For each sample, 0.25 g of rinsed and blotted-dry tissue was weighed, cut into small pieces, and transferred to a mortar. Following this, 2–3 mL of Anthocyan Assay Buffer was added, and the sample was ground thoroughly. The homogenate was transferred to a 10 mL centrifuge tube. The mortar was rinsed with additional buffer, and the rinse was combined with the homogenate. The total volume was adjusted to 8 mL using the same buffer, and the mixture was incubated at 4°C in the dark for 20 min. After centrifugation at 8,000 rpm for 3 min, the supernatant was collected as the crude anthocyanin extract. A 1–2 mL aliquot of the extract was transferred to a 1 cm cuvette, and absorbance was measured at 530 nm using a spectrophotometer, with the Anthocyan Assay Buffer as a blank. Three technical replicates were performed per sample, and three independent biological replicates (each composed of tissues pooled from 10 seedlings) were analyzed. The Anthocyan Assay Buffer was purchased from Suzhou Grace Biotechnology Co., Ltd (Zou et al., 2025). The anthocyanin content was calculated as:


$$\text{Anthocyanin}\left(\text{mg}/\text{g}\right)=\frac{\text{A}530\left(\text{measure}\right)-\text{A}530\left(\text{blank}\right)}{\text{sample weight}\left(\text{g}\right)}$$

### 5.5 Transcriptome sequencing and analysis

Total RNA was extracted from 0.5 g of leaf tissues using TRIzol reagent (Invitrogen, USA) following the manufacturer’s protocol (Lee, 2016). Genomic DNA contamination was removed by DNase I (Thermo Fisher Scientific) treatment. RNA integrity was verified by 1% agarose gel electrophoresis, and RNA concentration and purity were assessed using a NanoDrop ND-1000 spectrophotometer (A260/A280 ratio of 1.8–2.0).

For each sample, 3 μg of high-quality RNA was used for cDNA libraries construction with the NEBNext Ultra II RNA Library Prep Kit (NEB, USA). The library preparation included RNA fragmentation (300–500 bp), reverse transcription, adapter ligation, and PCR amplification (15 cycles). The resulting libraries were sequenced on an Illumina HiSeq X Ten platform (2 × 150 bp paired-end reads) at Novogene (Beijing, China), generating at least 30 Gb clean data per sample.

Raw reads were filtered to remove adapter sequences and low-quality reads (Q score< 20) using Trimmomatic v0.39 (Bolger et al., 2014). Clean reads were aligned to the *B. rapa* reference genome (v3.0) with HISAT2 v2.2.1, allowing up to two mismatches (Thakur, 2024). Gene expression levels were quantified with featureCounts v2.0.1 (Liao et al., 2014). DEGs were identified using DESeq2 v1.34.0 (Love et al., 2014), with thresholds of |log2FC| ≥ 1 and adjusted *P* < 0.05 (Benjamini-Hochberg correction).

GO enrichment analysis was performed using the clusterProfiler v4.2.2 package in R (Yu et al., 2012), with significance defined as *P* < 0.05 and FDR<0.05. KEGG pathway enrichment analysis was conducted against the KEGG database, and the results were visualized using TBtools v1.0987.

### 5.6 Hormone profiling

For quantification of ABA, GA, ACS, and SAM, enzyme-linked immunosorbent assays (ELISAs) were performed using commercial kits (Ruixinbio, Quanzhou, China) following the manufacturer’s protocols. Briefly, samples and standard solutions were added to 96-well microplates pre-coated with specific antibodies. After washing with PBS-Tween 20 (0.05%), horseradish peroxidase (HRP)-conjugated secondary antibodies were added, and the plates were incubated at 37°C for 30 min. Substrate solution (tetramethylbenzidine, TMB) was added to develop the color reaction, which was terminated with 2 M sulfuric acid. Absorbance was measured at 450 nm using a microplate reader (Multiskan FC, Thermo Fisher Scientific), and hormone concentrations were calculated from standard curves.

Total protein concentration in the extract was determined using a Bradford Protein Assay Kit (Sigma-Aldrich, USA) according to the manufacturer’s instructions, with bovine serum albumin (BSA) as a standard. Absorbance was measured at 595 nm, and protein concentration was calculated as mg/mL. Hormone concentrations were normalized to total protein content and expressed as ng/mg protein (ng/mg.pro). Each sample was assayed in triplicate, and data quality was validated by blank controls and spike recovery tests, with recovery rates ranging from 85% to 115%.

Statistical analysis was performed using one-way ANOVA followed by Duncan’s multiple range test (*P<* 0.05) in SPSS 26.0.

### 5.7 Gene expression validation

Quantitative real-time PCR (qRT-PCR) was performed for *BraA10g024990.3C* (*CHS*) using the SYBR Green Master Mix (Roche). Primers (*5’-ATGGTGATGGGTACACCGTC-3’* and *5’-GTCATGTGTTCGCTGTTGGT-3’*) were designed with Primer3. Reactions were run on a CFX96 Real-Time System (Bio-Rad), and gene expression was normalized to the *Actin* gene, with relative expression calculated by the 2^-(ΔΔCt)^ method (Livak and Schmittgen, 2001).

### 5.8 Bioinformatics analysis

CHS protein sequences were aligned using DNAMAN. Conserved domains (CHS/STS) were identified using the Pfam database (http://pfam.xfam.org/) (Bateman et al., 2004). Analysis of promoter cis-acting elements was performed using PlantCARE (http://bioinformatics.psb.ugent.be/webtools/plantcare/html/) (Lescot et al., 2002).

### 5.9 Exogenous hormone treatments

To investigate the regulatory effects of phytohormones on *CHS* expression and anthocyanin accumulation, exogenous hormone treatments were applied to four *B. rapa* inbred lines (129, 49, 16A-1 and R0-18). Seedlings at the four-leaf stage were sprayed with 100 µM ABA, 200 µM ethephon (an ethylene-releasing agent), or 200 µM GA3, based on previously reported concentrations in *B. rapa* (Bai et al., 2016; Thiruvengadam et al., 2015; Zheng et al., 2019). Distilled water sprayed on seedlings served as the control. Leaf samples were collected at 12 h post-treatment for RNA extraction and anthocyanin quantification. For each treatment, three independent biological replicates were performed, each consisting of 10 plants.

Total RNA was extracted and *CHS* expression was quantified by qRT-PCR as described above. Anthocyanin content was measured following the protocol described in the Anthocyanin quantification section. Statistical analysis was performed using one-way ANOVA followed by Duncan’s multiple range test (*P* < 0.05) in SPSS 26.0 to compare hormone treatments and genotypic differences.

### 5.10 Statistical analysis

Data were analyzed using one-way ANOVA followed by Duncan’s multiple range test (*P* < 0.05) in SPSS 26.0 (Permanasari and Santosa, 2010). Graphs were generated with GraphPad Prism 8.0.2 (Mitteer and Greer, 2022), and heatmaps were plotted using the pheatmap package in R (Kolde, 2025).

## Data Availability

The raw RNA-seq data generated in this study have been deposited in the NCBI Sequence Read Archive (SRA) under BioProject accession number PRJNA1480984.

## References

[B1] Arnon D. I. (1949). Copper enzymes in isolated chloroplasts. Polyphenoloxidase in Beta vulgaris. Plant Physiol. 24, 1–15. doi: 10.1104/pp.24.1.1 16654194 PMC437905

[B2] Bai Y. Zhu W. Hu X. Sun C. Li Y. Wang D. . (2016). Genome-wide analysis of the bZIP gene family identifies two ABI5-like bZIP transcription factors, BrABI5a and BrABI5b, as positive modulators of ABA signalling in Chinese cabbage. PLoS One 11, e0158966. doi: 10.1371/journal.pone.0158966 27414644 PMC4944949

[B3] Bano A. Ullah F. Nosheen A. (2022). Induction of salt tolerance in *Brassica rapa* by nitric oxide treatment. Front. Plant Sci. 13, 995837. doi: 10.3389/fpls.2022.995837 36466280 PMC9709477

[B4] Bateman A. Coin L. Durbin R. Finn R. D. Hollich V. Griffiths-Jones S. . (2004). Pfam: The protein families database. Nucleic Acids Res. 32, D138–D141. doi: 10.1093/nar/gkh121 14681378 PMC308855

[B5] Bolger A. M. Lohse M. Usadel B. (2014). Trimmomatic: A flexible trimmer for Illumina sequence data. Bioinformatics 30, 2114–2120. doi: 10.1093/bioinformatics/btu170 24695404 PMC4103590

[B6] Cao H. Li X. Chen Y. (2023). Alternative splicing control of light and temperature stress responses and its prospects in vegetable crops. Veg. Res. 3, 17. doi: 10.48130/vegres-2023-0017

[B7] Chen K. Li G. J. Bressan R. A. Song C. P. Zhu J. K. Zhao Y. (2020). Abscisic acid dynamics, signaling, and functions in plants. J. Integr. Plant Biol. 62, 25–54. doi: 10.1111/jipb.12899 31850654

[B8] Dabravolski S. A. Isayenkov S. V. (2023). The role of anthocyanins in plant tolerance to drought and salt stresses. Plants 12, 2558. doi: 10.3390/plants12132558 37447119 PMC10346810

[B9] Di Ferdinando M. Brunetti C. Fini A. Tattini M. (2012). “ Flavonoids as antioxidants in plants under abiotic stresses,” in Abiotic Stress Responses in Plants: Metabolism, Productivity and Sustainability. Eds. Ahmad P. Prasad M. N. V. ( Springer, New York, NY), 159–179. doi: 10.1007/978-1-4614-0634-19

[B10] Ferreyra M. L. F. Rius S. P. Casati P. (2021). Recent advances on the roles of flavonoids as plant protective molecules after UV and high light exposure. Physiol. Plant 173, 736–749. doi: 10.1111/ppl.13543 34453749

[B11] Guo M. Liu J. H. Ma X. Luo D. X. Gong Z. H. Lu M. H. (2016). The plant heat stress transcription factors (HSFs): Structure, regulation, and function in response to abiotic stresses. Front. Plant Sci. 7, 114. doi: 10.3389/fpls.2016.00114 26904076 PMC4746267

[B12] Higashi Y. Saito K. (2019). Lipidomic studies of membrane glycerolipids in plant leaves under heat stress. Prog. Lipid Res. 75, 100990. doi: 10.1016/j.plipres.2019.100990 31442527

[B13] Irulappan V. Thomas J. Mohanapriya A. Senthil-Kumar M. (2023). Genome-wide identification of a novel Na+ transporter from *Bienertia sinuspersici* and overexpression of BsHKT1;2 improved salt tolerance in *Brassica rapa*. Front. Plant Sci. 14, 1302315. doi: 10.3389/fpls.2023.1302315 38192689 PMC10773568

[B14] Johnson S. E. Rieseberg L. H. (2022). Rapid‐cycling *Brassica rapa* evolves even earlier flowering under experimental drought. Am. J. Bot. 109, 1683–1692. doi: 10.1002/ajb2.16087 35587234

[B15] Kan Y. Mu X. R. Zhang H. Gao J. Shan J. X. Ye W. W. . (2023). The molecular basis of heat stress responses in plants. Mol. Plant 16, 1612–1634. doi: 10.1016/j.molp.2023.09.013 37740489

[B16] Kolde R. (2025). Pheatmap: Pretty Heatmaps. R Package Version 1.0.13. Available online at: https://github.com/raivokolde/pheatmap.

[B17] Lamers J. van der Meer T. Testerink C. (2025). Abscisic acid signaling gates salt-induced responses of plant roots. Proc. Natl. Acad. Sci. U.S.A. 122, e2406373122. doi: 10.1073/pnas.2406373122 39908104 PMC11831169

[B18] Laxa M. Liebthal M. Telman W. Chibani K. Dietz K. J. (2019). The role of the plant antioxidant system in drought tolerance. Antioxidants 8, 94. doi: 10.3390/antiox8040094 30965652 PMC6523806

[B19] Lee O. R. (2016). In silico analysis of MeJA-induced comparative transcriptomes in *Brassica oleraceae* L. var. *capitata*. J. Plant Biotechnol. 43, 189–203. doi: 10.5010/jpb.2016.43.2.189

[B20] Lee S. C. Lim M. H. Kim J. A. Lee S. I. Kim J. S. Jin M. . (2008). Transcriptome analysis in *Brassica rapa* under the abiotic stresses using Brassica 24K oligo microarray. Mol. Cells 26, 595–605. doi: 10.1016/s1016-8478(23)14042-8 18797175

[B21] Lescot M. Déhais P. Thijs G. Marchal K. Moreau Y. Van de Peer Y. . (2002). PlantCARE, a database of plant cis-acting regulatory elements and a portal to tools for in *silico* analysis of promoter sequences. Nucleic Acids Res. 30, 325–327. doi: 10.1093/nar/30.1.325 11752327 PMC99092

[B22] Li S. Fu Q. Chen L. Huang W. Yu D. (2010). Functional characterization of Arabidopsis thaliana WRKY39 in heat stress. Mol. Cells 29, 475–484. doi: 10.1007/s10059-010-0059-2 20396965

[B23] Liao Y. Smyth G. K. Shi W. (2014). featureCounts: An efficient general purpose program for assigning sequence reads to genomic features. Bioinformatics 30, 923–930. doi: 10.1093/bioinformatics/btt656 24227677

[B24] Liu H. Song S. Zhang H. (2022). Signaling transduction of ABA, ROS, and Ca^2+^ in plant stomatal closure in response to drought. Int. J. Mol. Sci. 23, 14824. doi: 10.3390/ijms232314824 36499153 PMC9736234

[B25] Liu J. X. Srivastava R. Che P. Howell S. H. (2007). Salt stress responses in Arabidopsis utilize a signal transduction pathway related to endoplasmic reticulum stress signaling. Plant J. 51, 897–909. doi: 10.1111/j.1365-313X.2007.03195.x 17662035 PMC2156172

[B26] Livak K. J. Schmittgen T. D. (2001). Analysis of relative gene expression data using real-time quantitative PCR and the 2-ΔΔCT method. Methods 25, 402–408. doi: 10.1006/meth.2001.1262 11846609

[B27] Love M. I. Huber W. Anders S. (2014). Moderated estimation of fold change and dispersion for RNA-seq data with DESeq2. Genome Biol. 15, 550. doi: 10.1186/s13059-014-0550-8 25516281 PMC4302049

[B28] Mishra G. Zhang W. Deng F. Zhao J. Wang X. (2006). A bifurcating pathway directs abscisic acid effects on stomatal closure and opening in Arabidopsis. Science 312, 264–266. doi: 10.1126/science.1123769 16614222

[B29] Mitteer D. R. Greer B. D. (2022). Using GraphPad Prism's heat maps for efficient, fine-grained analyses of single-case data. Behav. Anal. Pract. 15, 505–514. doi: 10.1007/s40617-021-00664-7 35692516 PMC9120324

[B30] Mondal S. Singh A. (2023). Crucial plant processes under heat stress and tolerance through heat shock proteins. Plant Stress 10, 100227. doi: 10.1016/j.stress.2023.100227 38826717

[B31] Muthusamy M. Kim J. H. Yoon E. K. Kim J. A. Lee S. I. (2020). Brassica rapa SR45a regulates drought tolerance via the alternative splicing of target genes. Genes 11, 182. doi: 10.3390/genes11020182 32050656 PMC7074037

[B32] Ohama N. Sato H. Shinozaki K. Yamaguchi-Shinozaki K. (2017). Transcriptional regulatory network of plant heat stress response. Trends Plant Sci. 22, 53–65. doi: 10.1016/j.tplants.2016.10.004 27666516

[B33] Patil J. R. Sharma A. (2024). Flavonoids in plant-environment interactions and stress responses. Discov. Plants 1, 68. doi: 10.1007/s44372-024-00063-6 30311153

[B34] Pearson K. (1895). Notes on regression and inheritance in the case of two parents. Proc. R. Soc Lond. 58, 240–242. doi: 10.1098/rspl.1895.0041 34341189

[B35] Permanasari A. Santosa B. (2010). “ Forecasting method selection using ANOVA and Duncan multiple range tests on time series dataset”, in: 2010 International Symposium on Information Technology. Kuala Lumpur: IEEE. 2, 941–945. doi: 10.1109/ITSIM.2010.5561535

[B36] Quan J. Zhang L. Wang Y. Li Z. (2023). Transcriptome of heat stress response in Brassica rapa L. ssp. pekinensis with improved thermotolerance through exogenous glycine betaine. Int. J. Mol. Sci. 24, 6429. doi: 10.3390/ijms24076429 37047402 PMC10094913

[B37] Ren Y. Wang Y. Zhang L. (2021). A heat stress responsive NAC transcription factor heterodimer plays key roles in rice grain filling. J. Exp. Bot. 72, 2947–2964. doi: 10.1093/jxb/erab027 33476364

[B38] Reynolds T. W. Waddington S. R. Anderson C. L. Chew A. True Z. Cullen A. (2015). Environmental impacts and constraints associated with the production of major food crops in Sub-Saharan Africa and South Asia. Food Secur. 7, 795–822. doi: 10.1007/s12571-015-0478-1 30311153

[B39] Sun W. Liang L. (2019). Chalcone isomerase a key enzyme for anthocyanin biosynthesis in *Ophiorrhiza japonica*. Front. Plant Sci. 10, 865. doi: 10.3389/fpls.2019.00865 31338101 PMC6629912

[B40] Ta Na Zhao W. G. Shi Y. X. Gao H. L. Li C. J. Wang H. . (2026). Effects of simulated drought stress on related traits and physiological indicators of KN DH population in *Brassica napus*. Acta Agric. Jiangxi 38, 58–65. doi: 10.19386/j.cnki.jxnyxb.2026.04.008

[B41] Thakur V. (2024). RNA-Seq data analysis for differential gene expression using HISAT2–StringTie–Ballgown pipeline. Methods Mol. Biol. 2812, 101–113. doi: 10.1007/978-1-0716-3886-6_5 39068358

[B42] Thiruvengadam M. Kim S. H. Chung I. M. (2015). Exogenous phytohormones increase the accumulation of health-promoting metabolites, and influence the expression patterns of biosynthesis related genes and biological activity in Chinese cabbage (*Brassica rapa* spp. *pekinensis*). Sci. Hortic. 193, 136–146. doi: 10.1016/j.scienta.2015.07.007 38826717

[B43] Timoneda A. Feng T. Sheehan H. Walker-Hale N. Pucker B. Lopez-Nieves S. . (2019). The evolution of betalain biosynthesis in Caryophyllales. New Phytol. 224, 71–85. doi: 10.1111/nph.15980 31172524

[B44] Verma V. Ravindran P. Kumar P. P. (2016). Plant hormone-mediated regulation of stress responses. BMC Plant Biol. 16, 86. doi: 10.1186/s12870-016-0771-y 27079791 PMC4831116

[B45] Waadt R. Seller C. A. Hsu P. K. Takahashi Y. Munemasa S. Schroeder J. I. (2022). Plant hormone regulation of abiotic stress responses. Nat. Rev. Mol. Cell Biol. 23, 680–694. doi: 10.1038/s41580-022-00479-6 35513717 PMC9592120

[B46] Wang X. Mao Z. Zhang J. Hemat M. Huang M. Cai J. . (2019). Osmolyte accumulation plays important roles in the drought priming induced tolerance to post-anthesis drought stress in winter wheat (*Triticum aestivum* L.). Environ. Exp. Bot. 166, 103804. doi: 10.1016/j.envexpbot.2019.103804 38826717

[B47] Wang Y. Zhang L. Li Z. (2024). Two lncRNAs of Chinese cabbage confer Arabidopsis with heat and drought tolerance. Veg. Res. 4, e029. doi: 10.48130/vegres-0024-0029

[B48] Xu X. Zhang Y. Wang P. (2025). Stomatal opening under high temperatures is controlled by the OST1-regulated TOT3–AHA1 module. Nat. Plants 11, 105–117. doi: 10.1038/s41477-024-01859-w 39613896

[B49] Yamada K. Fukao Y. Hayashi M. Fukazawa M. Suzuki I. Nishimura M. (2007). Cytosolic HSP90 regulates the heat shock response that is responsible for heat acclimation in *Arabidopsis thaliana*. J. Biol. Chem. 282, 37794–37804. doi: 10.1074/jbc.M707168200 17965410

[B50] Yang Y. Guo Y. (2017). Elucidating the molecular mechanisms mediating plant salt‐stress responses. New Phytol. 217, 523–539. doi: 10.1111/nph.14920 29205383

[B51] Yu B. Li Z. (2024). How plants sense and respond to osmotic stress. J. Integr. Plant Biol. 66, 394–423. doi: 10.1111/jipb.13617 38329193

[B52] Yu G. Wang L. G. Han Y. He Q. Y. (2012). clusterProfiler: An R package for comparing biological themes among gene clusters. OMICS 16, 284–287. doi: 10.1089/omi.2011.0118 22455463 PMC3339379

[B53] Zandalinas S. I. Mittler R. (2022). Plant responses to multifactorial stress combination. New Phytol. 234, 1161–1167. doi: 10.1111/nph.18087 35278228

[B54] Zhang L. Dai Y. Yue L. Wang J. Li Z. (2022). Heat stress response in Chinese cabbage (*Brassica rapa* L.) revealed by transcriptome and physiological analysis. PeerJ 10, e13427. doi: 10.7717/peerj.13427 35637719 PMC9147330

[B55] Zhang W. Liu Y. Wang J. Chen X. (2023). H_2_S-mediated balance regulation of stomatal and non-stomatal factors responding to drought stress in Chinese cabbage. Hortic. Res. 10, uhac284. doi: 10.1093/hr/uhac284 36938567 PMC10018781

[B56] Zheng H. Li X. Shi L. Jing Y. Song Q. Chen Y. . (2019). Genome-wide identification and analysis of the cytochrome B5 protein family in Chinese cabbage (*Brassica rapa* L. ssp. *Pekinensis*). Int. J. Genomics 2019, 2102317. doi: 10.1155/2019/2102317 31871927 PMC6913312

[B57] Zheng X. Li Y. Zhang H. (2025). The regulatory network composed of phytohormones, transcription factors and non-coding RNAs is involved in the flavonoids biosynthesis of fruits. Hortic. Plant J. 12, 497–508. doi: 10.1016/j.hpj.2024.05.008 38826717

[B58] Zou Q. Bao T. Yu L. Xu H. Liu W. Li Z. . (2025). The regulatory module MdCPCL-MdILR3L mediates the synthesis of ascorbic acid and anthocyanin in apple. Plant Biotechnol. J. 23, 1101–1117. doi: 10.1111/pbi.14567 39777958 PMC11933874

