# Supplementary material for: Integrated transcriptomic and hormonal analysis suggests a trade-off between salt/drought tolerance and thermotolerance in Brassica rapa
Source: Front Plant Sci. 2026 Jul 27;17:1868194. doi: 10.3389/fpls.2026.1868194 (PMC13455000; doi:10.3389/fpls.2026.1868194)
Supplement: Supplementary file 1 [file Supplementaryfile1.pdf]

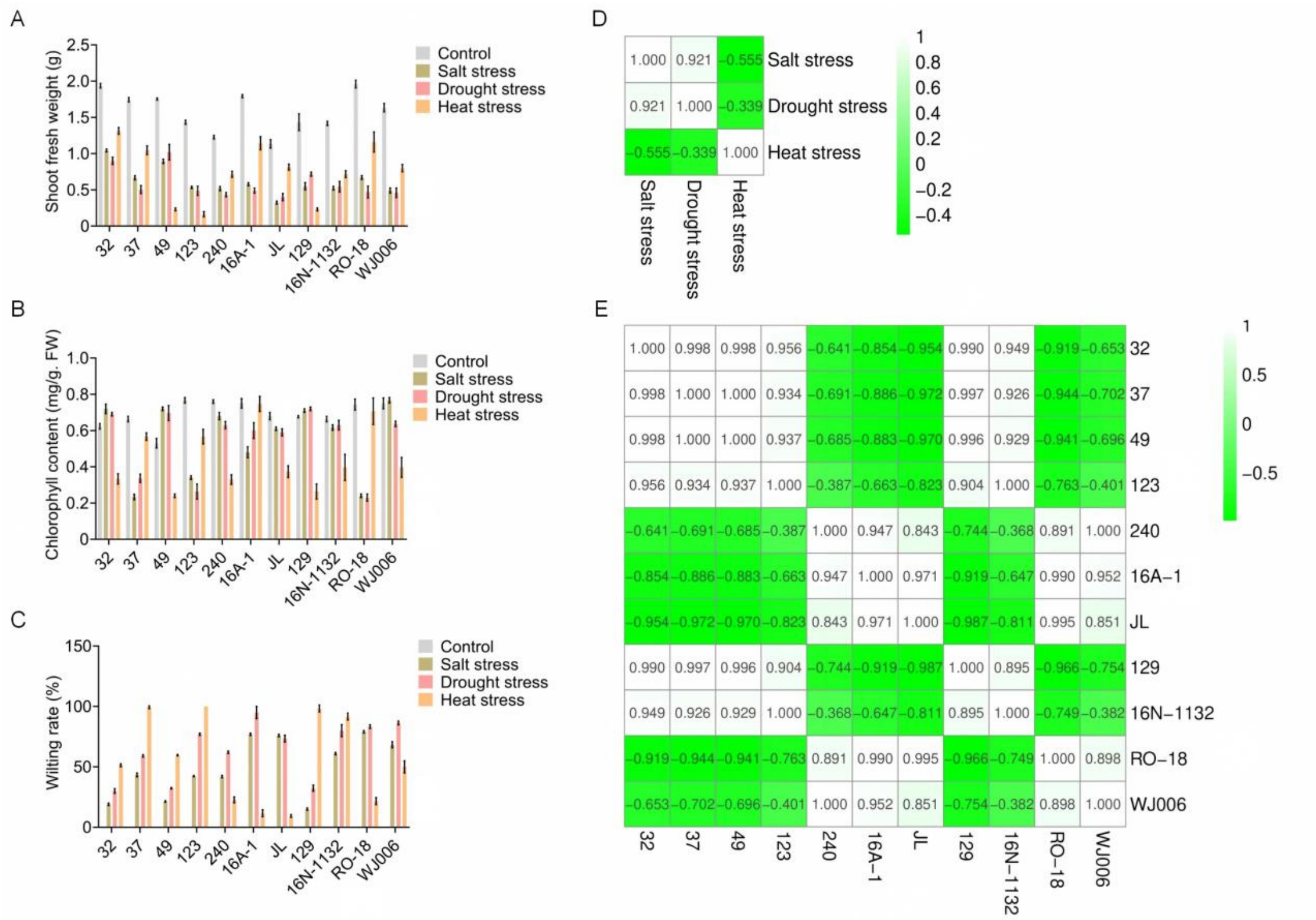

Fig. S1. Physiological evaluation of 11 *B. rapa* inbred lines under salt, drought, and heat stresses.

(A) Shoot fresh weight. (B) Chlorophyll content. (C) Wilting rate. (D) Pearson correlation analysis among salt injury index, drought injury index and heat injury index. Correlation coefficients (r) are shown, with color intensity of the squares indicating the strength of correlation. (E) Pearson correlation analysis among salt tolerance, drought tolerance and heat tolerance across the 11 lines. The correlation matrix was used to cluster the lines into two distinct groups based on their integrated stress tolerance profiles: Group I (thermotolerant: 240, 16A-1, JL, R0-18 and WJ006) and Group II (salt/drought tolerant: 32, 37, 49, 123, 129 and 16N-1132). Data are presented as mean ± SD (n = 3 biological replicates, each with 10 plants).

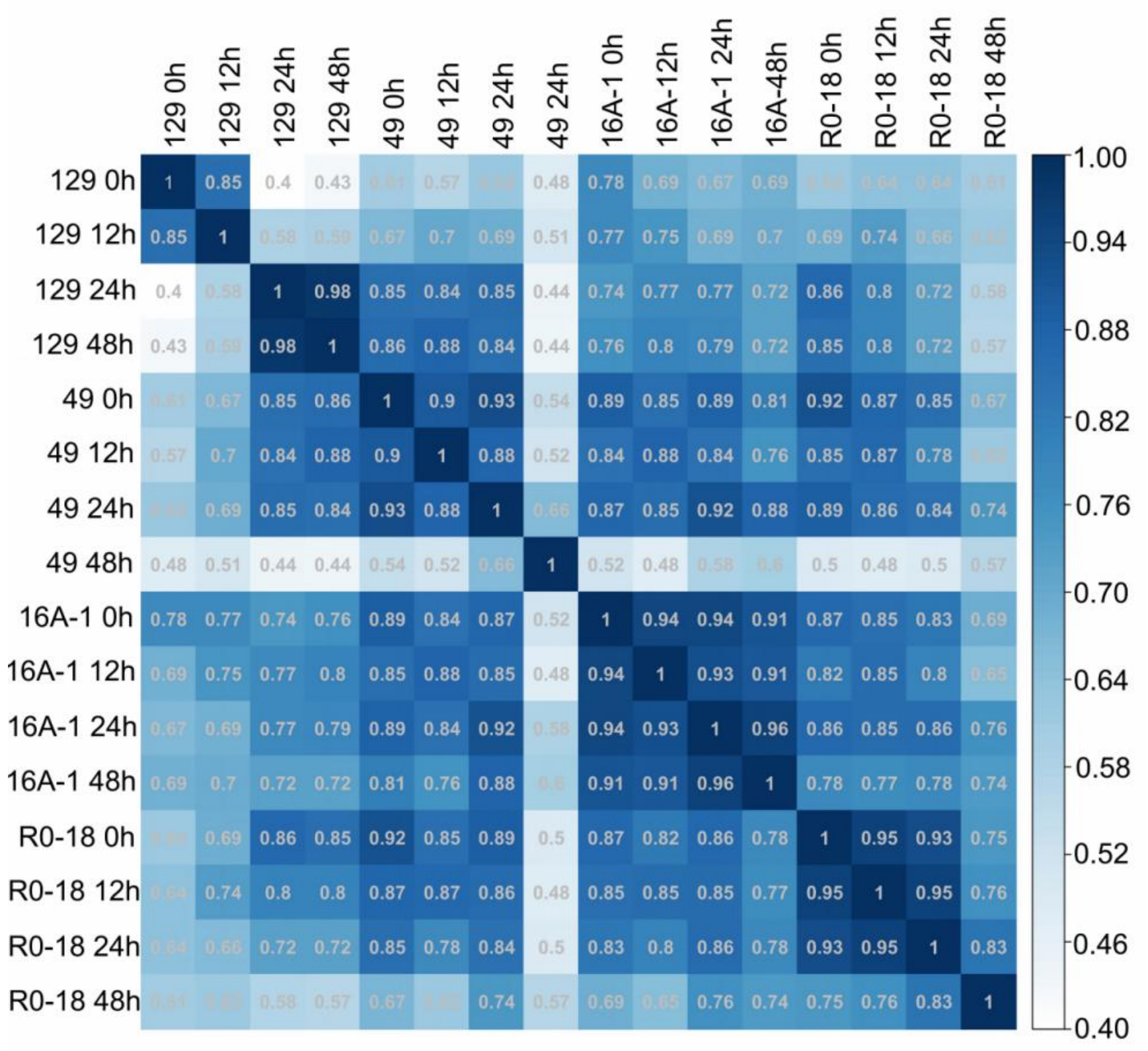

Fig. S2. Pearson correlation heatmap of transcriptomes under salt stress.

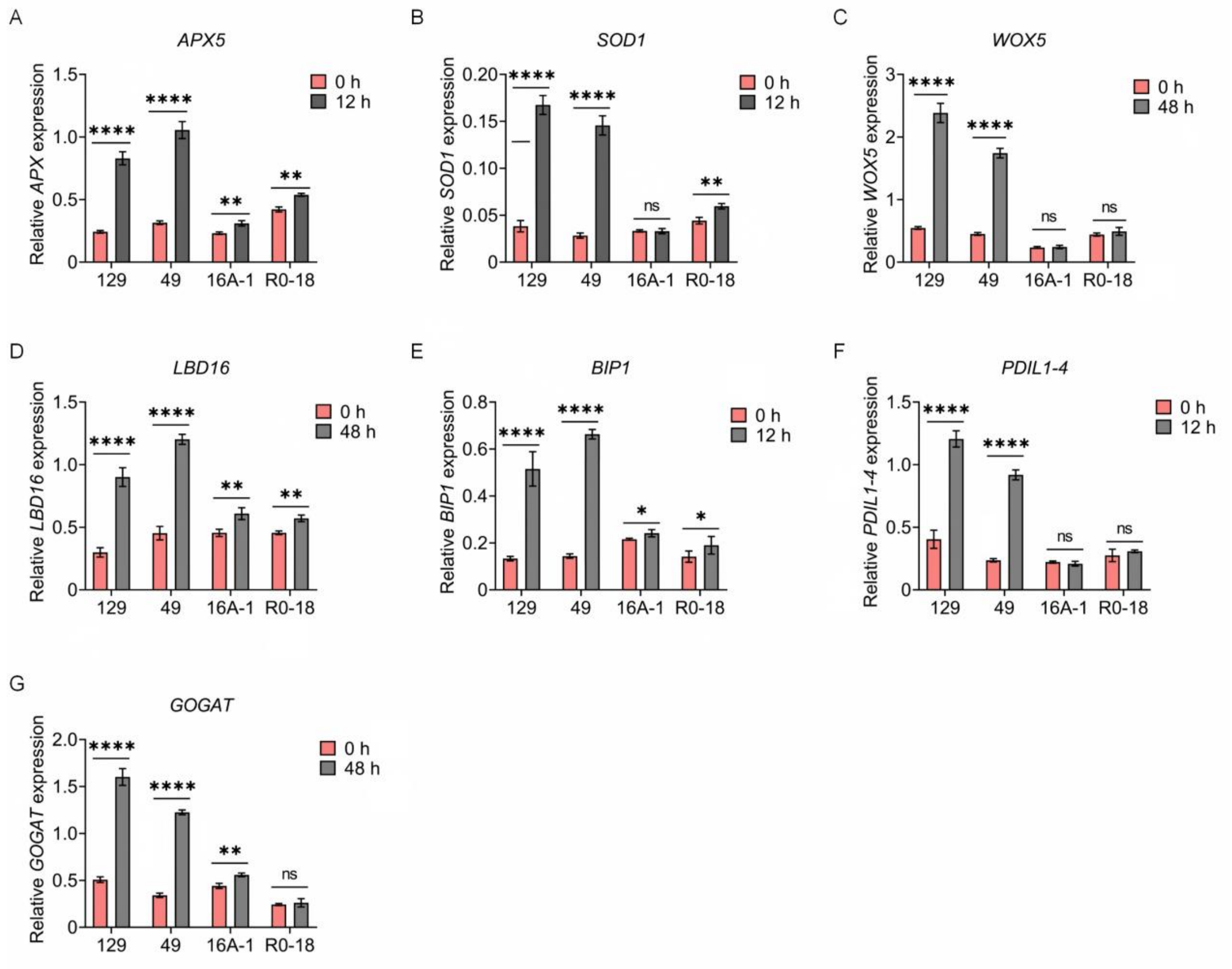

Fig. S3. Expression profiles of key salt tolerance-associated genes in response to salt stress.

Transcript levels of *APX5, SOD1, BiP1, PDIL1-4, WOX5, LBD16* and *GOGAT* were quantified by qRT-PCR in the four genotypes (129, 49, 16A-1 and R0-18) at 0 and 48 h after 250 mM NaCl treatment. Values are presented as means ± SD of three independent biological replicates. Asterisks indicate significant differences compared to the corresponding control (0 h) at the same time point (* $P < 0.05$; **$P < 0.01$, ****$P < 0.0001$,, Student's t-test). Gene expression was normalized to the internal reference gene *BraActin*. Note: *APX5,* ascorbate peroxidase 5; *SOD1,* superoxide dismutase 1; *BiP1,* binding immunoglobulin protein 1; *PDIL1-4,* protein disulfide isomerase-like 1-4; *WOX5,* WUSCHEL-related homeobox 5; *LBD16,* lateral organ boundaries domain 16; *GOGAT,* glutamate synthase.

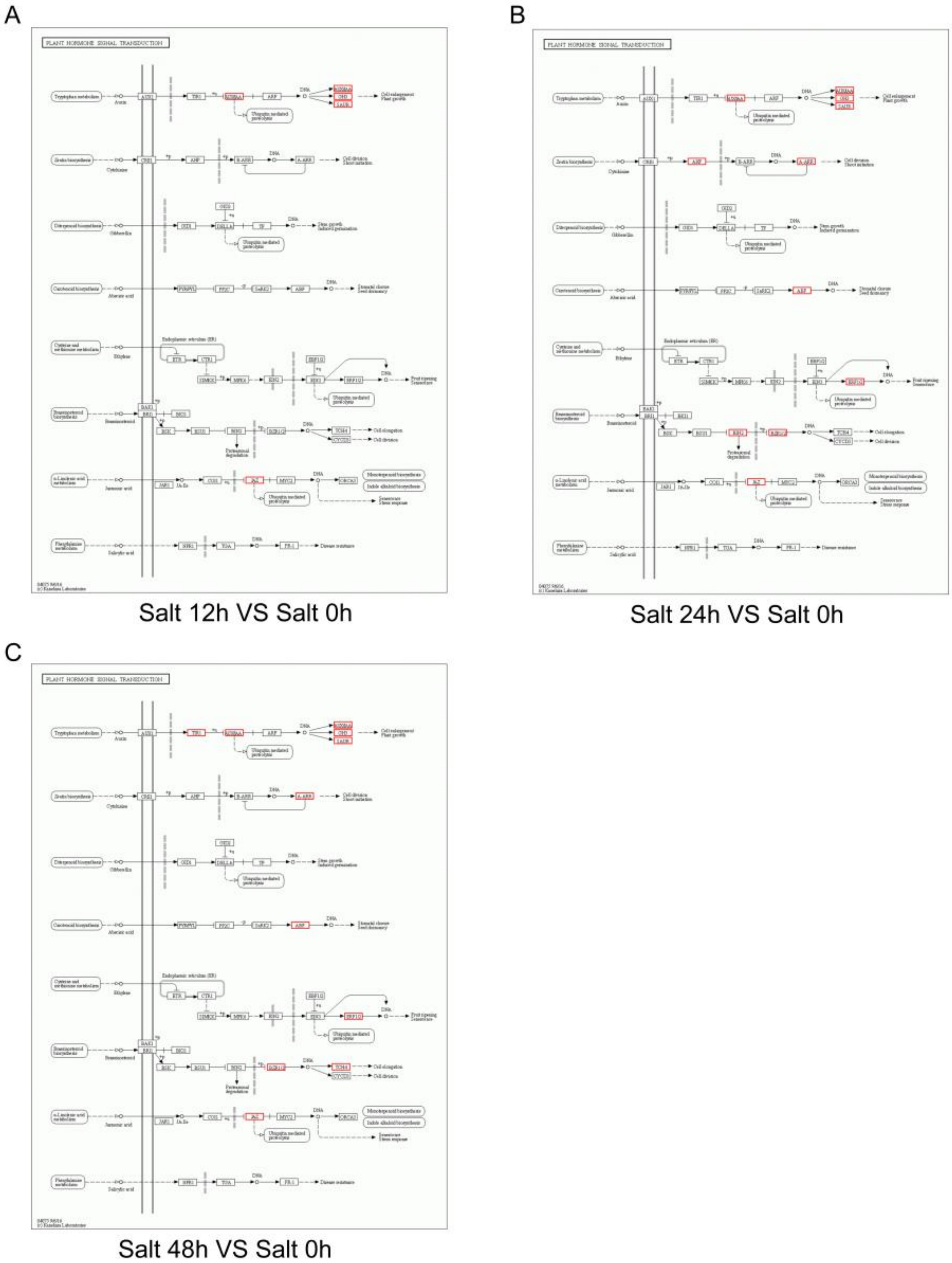

Fig. S4. KEGG enrichment of hormone signaling in salt-tolerant lines at 12 h (A), 24 h (B), and 48 h (C).

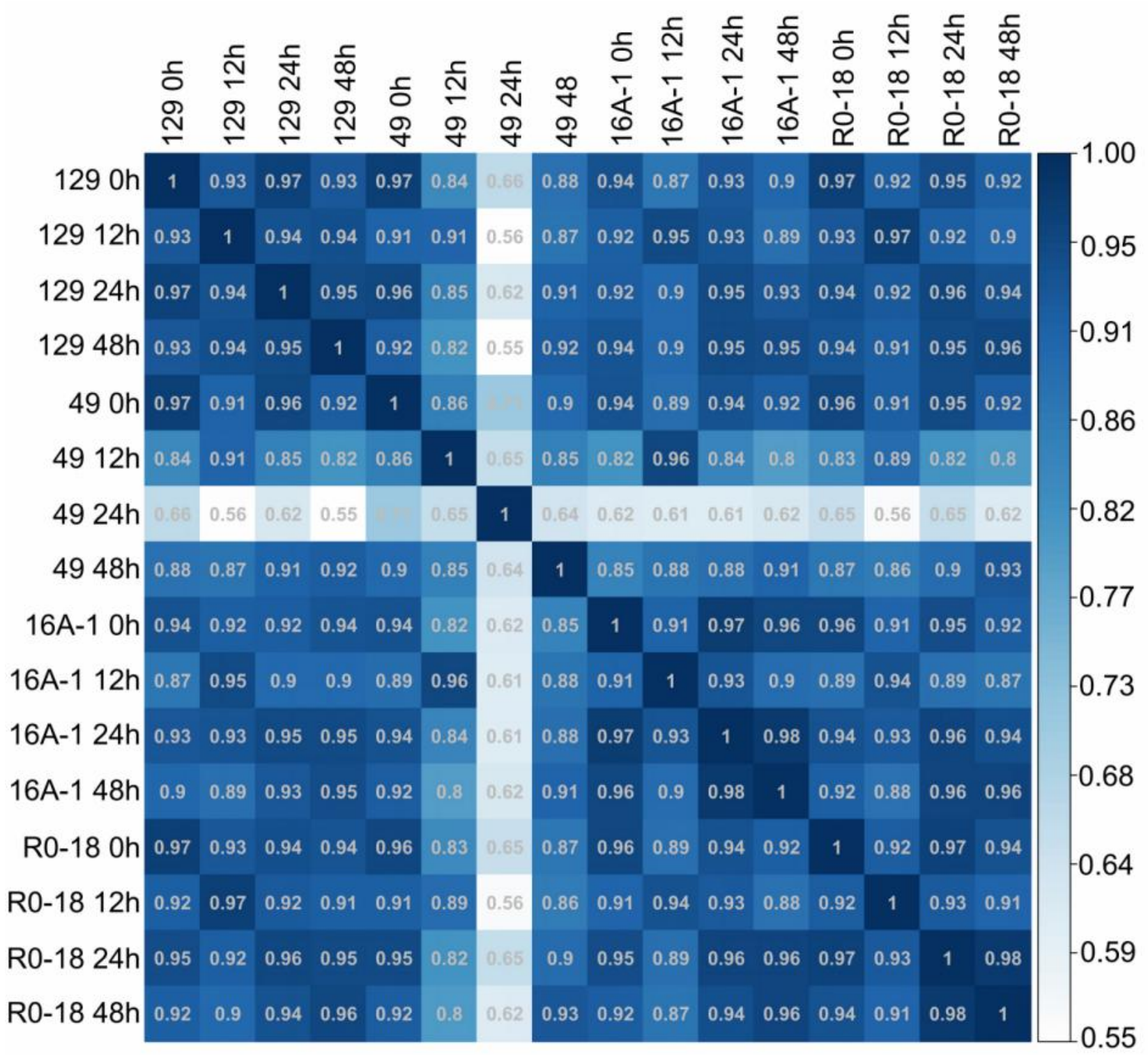

Fig. S5. Pearson correlation heatmap of transcriptomes under drought stress.

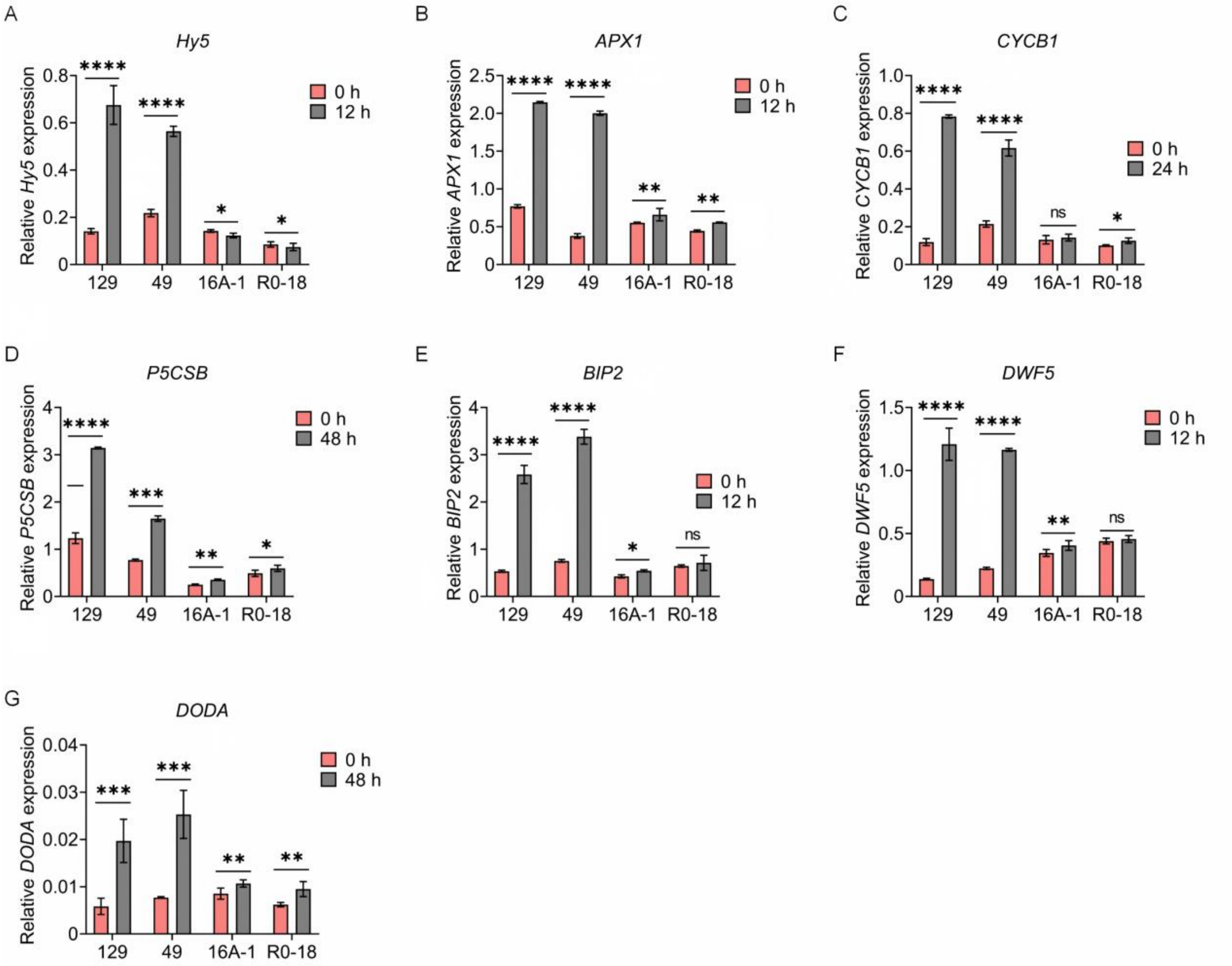

Fig. S6. Expression profiles of key drought-associated genes in response to drought stress.

Transcript levels of *HY5, APX1, BiP2, DWF5, CYCB1, P5CSB* and *DODA* were quantified by qRT-PCR in the four genotypes (129, 49, 16A-1 and R0-18) at 0 and 48 h after 20% PEG6000 treatment. Values are presented as means ± SD of three independent biological replicates. Asterisks indicate significant differences compared to the corresponding control (0 h) at the same time point (* P < 0.05; **P < 0.01, ***P < 0.001, ****P < 0.0001, Student's t-test). Gene expression was normalized to the internal reference gene *BraActin*. Note: *HY5,* elongated hypocotyl 5; *APX1,* ascorbate peroxidase 1; *BiP2,* binding immunoglobulin protein 2; *DWF5,* dwarf 5; *CYCB1,* cyclin B1; P5CSB, Δ^1-pyrroline-5-carboxylate synthetase B; *DODA,* dopa 4,5-dioxygenase.

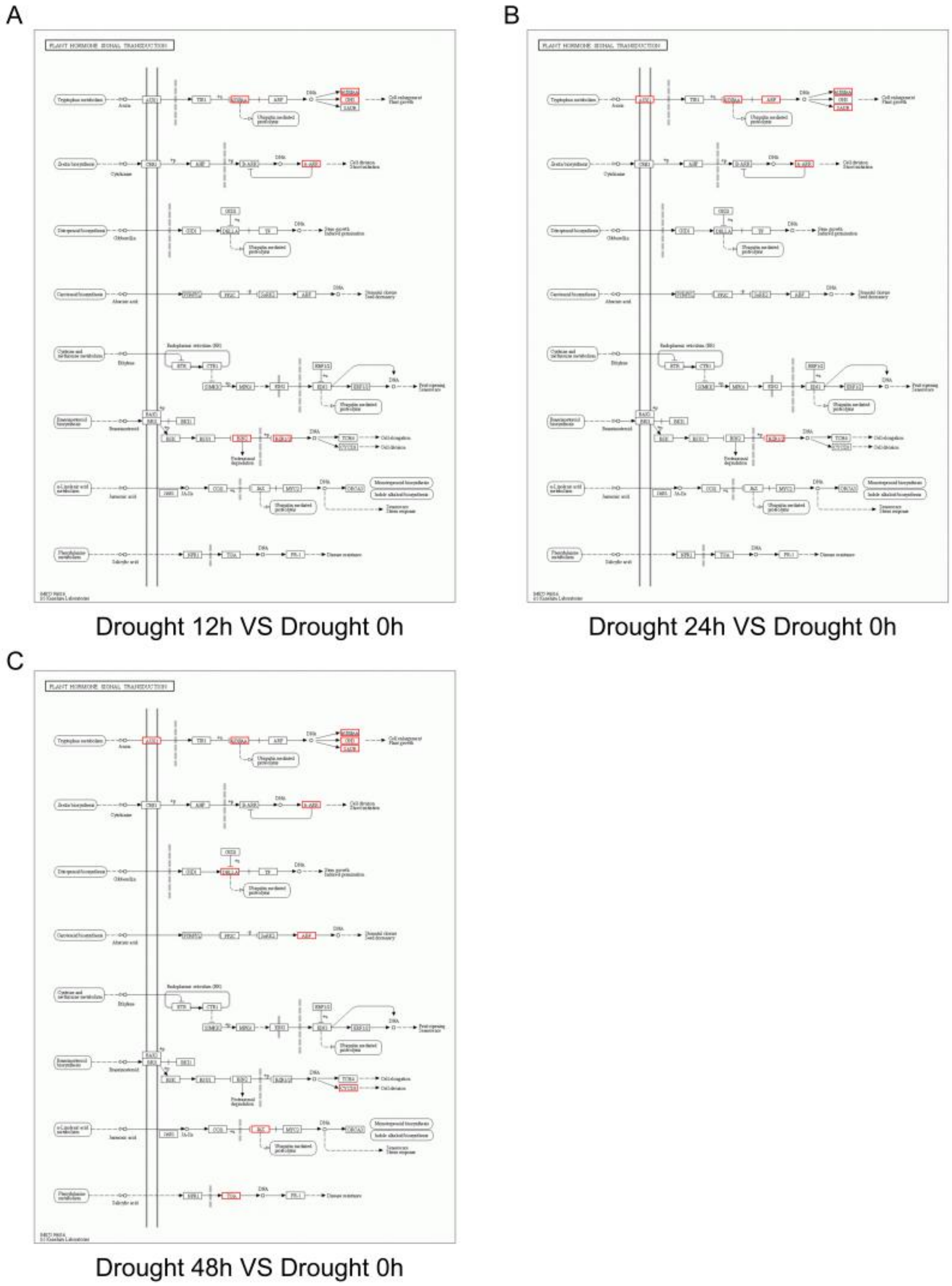

Fig. S7. KEGG enrichment of hormone signaling in drought-tolerant lines at 12 h (A), 24 h (B), and 48 h (C).

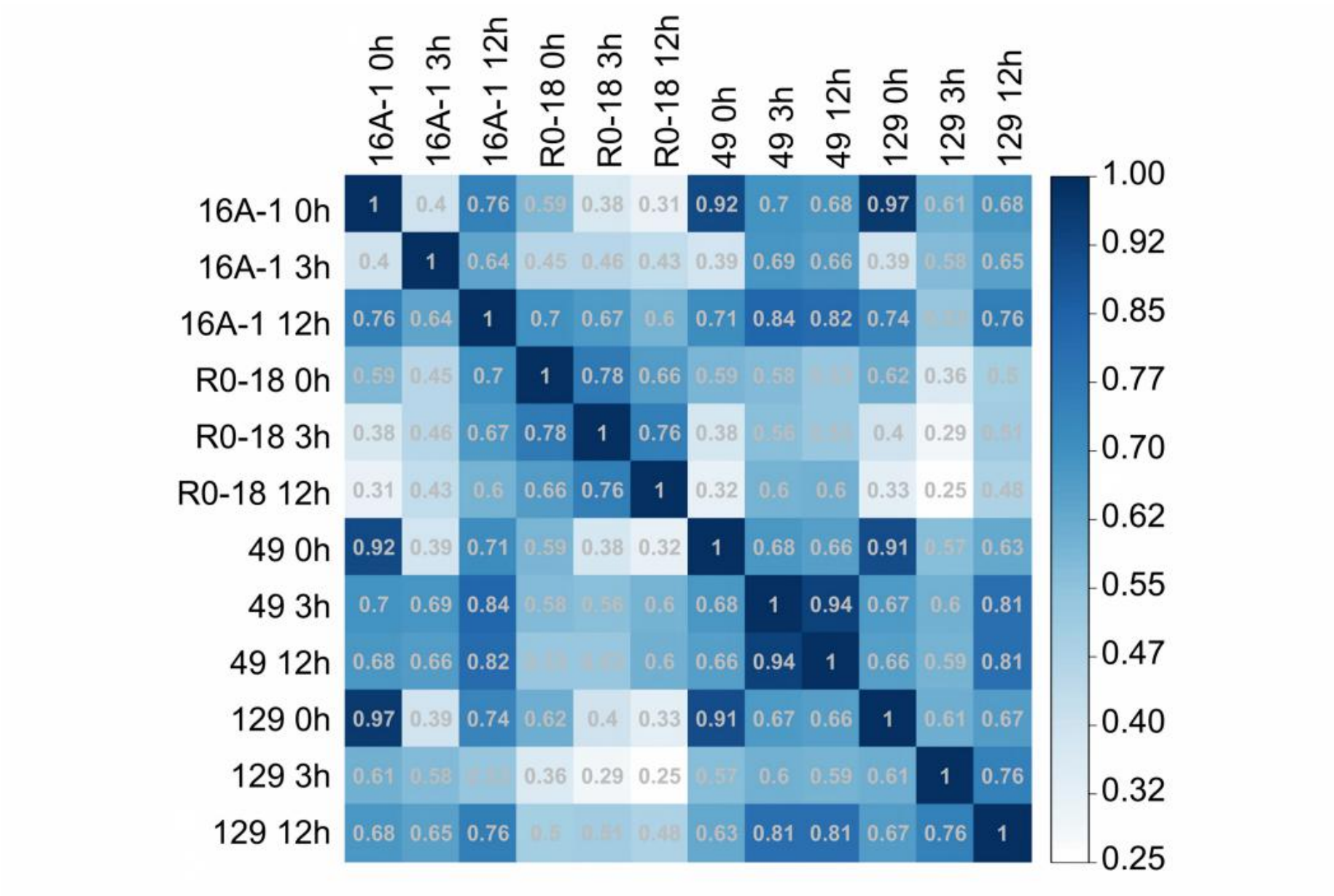

Fig. S8. Pearson correlation heatmap of transcriptomes under heat stress.

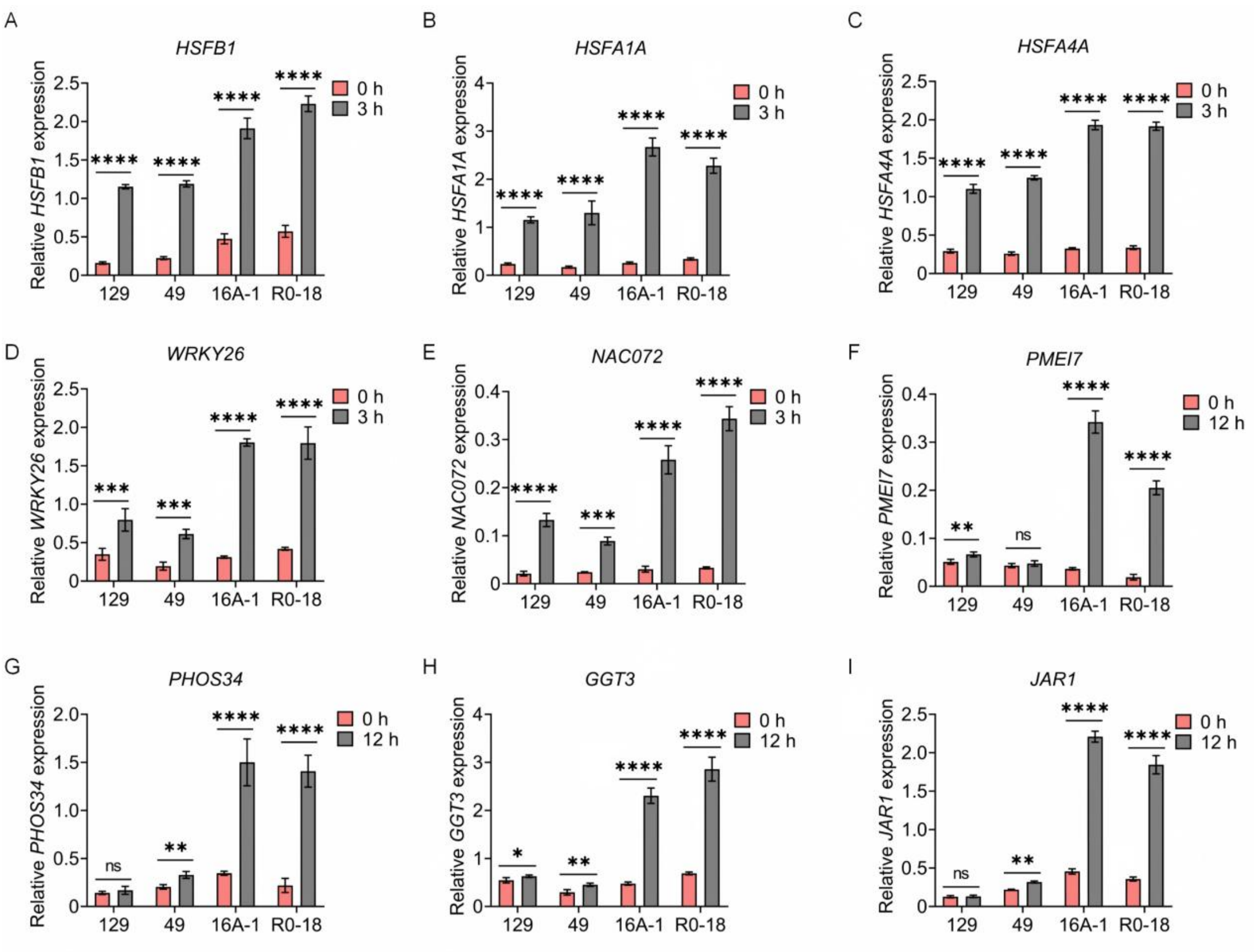

Fig. S9. Expression profiles of key thermotolerance-associated genes in response to heat stress.

Transcript levels of *HSFB1, HSFA4A, WRKY26, NAC072, PMEI7, PHOS34, GGT3,* and *JAR1* were quantified by qRT-PCR in the four genotypes (129, 49, 16A-1 and R0-18) at 0 and 12 h after 40°C heat stress. Values are presented as mean ± SD of three independent biological replicates. Asterisks indicate significant differences compared to the corresponding control (0 h) at the same time point (* $P < 0.05$; **$P < 0.01$, ***$P < 0.001$, ****$P < 0.0001$, Student's t-test). Gene expression was normalized to the internal reference gene *BraActin*. Note: *HSFB1,* heat shock factor B1; *HSFA4A,* heat shock factor A4A; *WRKY26,* WRKY transcription factor 26; *NAC072,* NAC domain-containing protein 72; *PMEI7,* pectin methylesterase inhibitor 7; *PHOS34,* phosphate transporter 34; *GGT3,* gamma-glutamyltransferase 3; *JAR1,* jasmonic acid-amino synthetase 1.

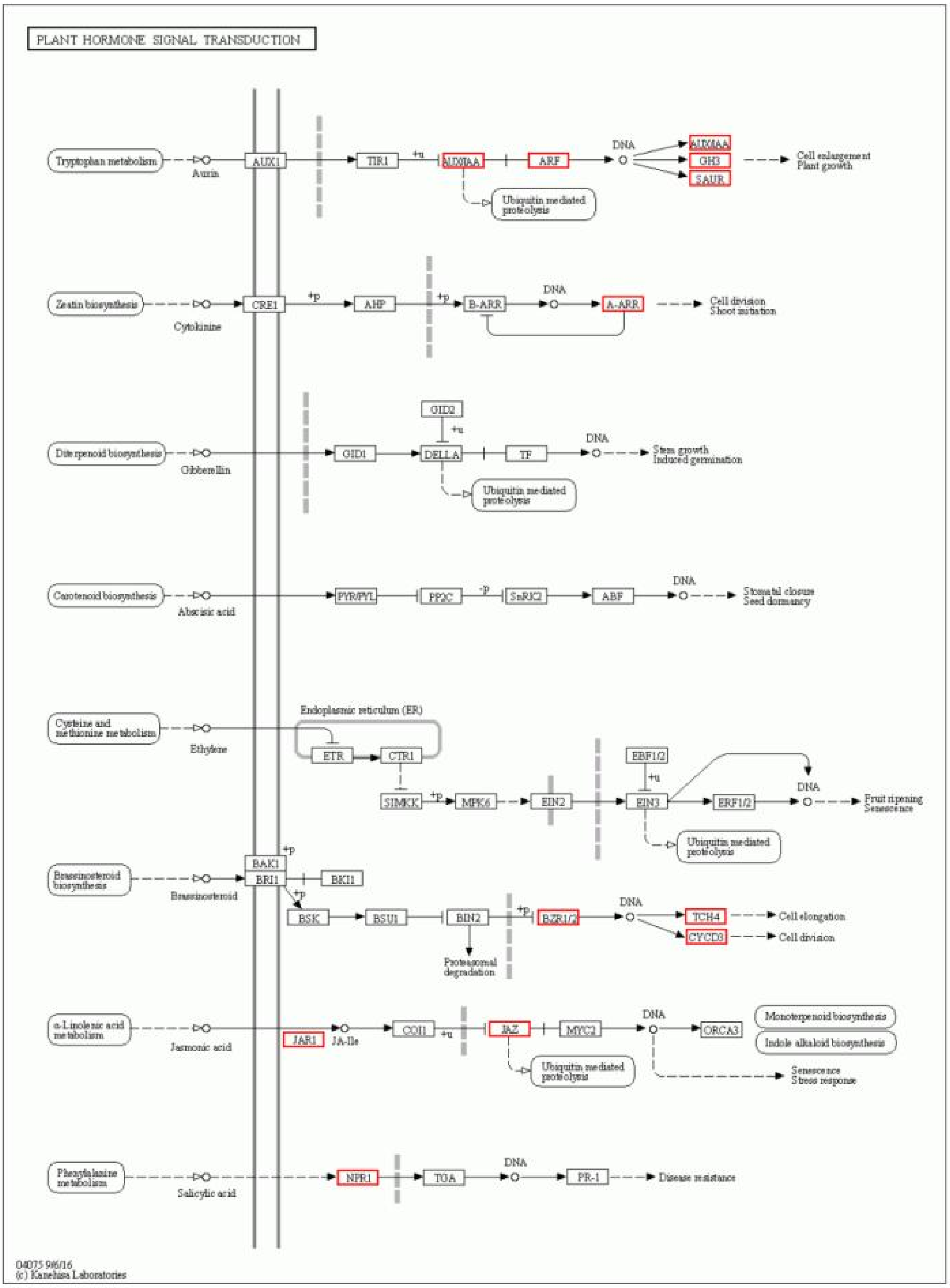

Heat 12h vs Heat 0h

Fig. S10. KEGG enrichment of hormone signaling in thermotolerant lines at 12 h.

>BraA10g024990.3C

MVMGTPSSLDEIRKAQRADGPAGILAIGTANPANHVIQAEYPDYYFRITNSE

HMTDLKEKFKRMCDKSTIRKRHMHLTEEFLKENPDMCAYMAPSLDARQDIV

PF00195 Chalcone and stilbene synthases, N-terminal domain

VVEVPKLGKEAAVKAIKEWGQPKSKITHVVFCTTSGVDMPGADYQLTKLLG

LRPSVKRLMMYQQGCFAGGTVLRLAKDLAENNRGARVLVVCSEITAVTFRG

PSDTHLDSLVGQALFSDGAAALIVGSDADTSAGEKPIFEMVSAAQTILPDSD

GAIDGHLREVGLTFHLLKDVPGLISKNIEKSLDEAFKPLGISDWNSLFWIAHP

PF02797 Chalcone and stilbene synthases, C-terminal domain

GGPAILDEVEKKLGLKAEKMRATRHVLSEYGNMSSACVLFILDEMRRKSAE

DGVATTGEGLEWGVLFGFGPGLTVETVVLHSVPV

Fig. S11. Conserved domains of the BraA10g024990.3C (CHS) protein.

The N-terminal chalcone and stilbene synthases domain (PF00195) is highlighted in blue, and the C-terminal domain (PF02797) is highlighted in yellow, as identified by Pfam database analysis.

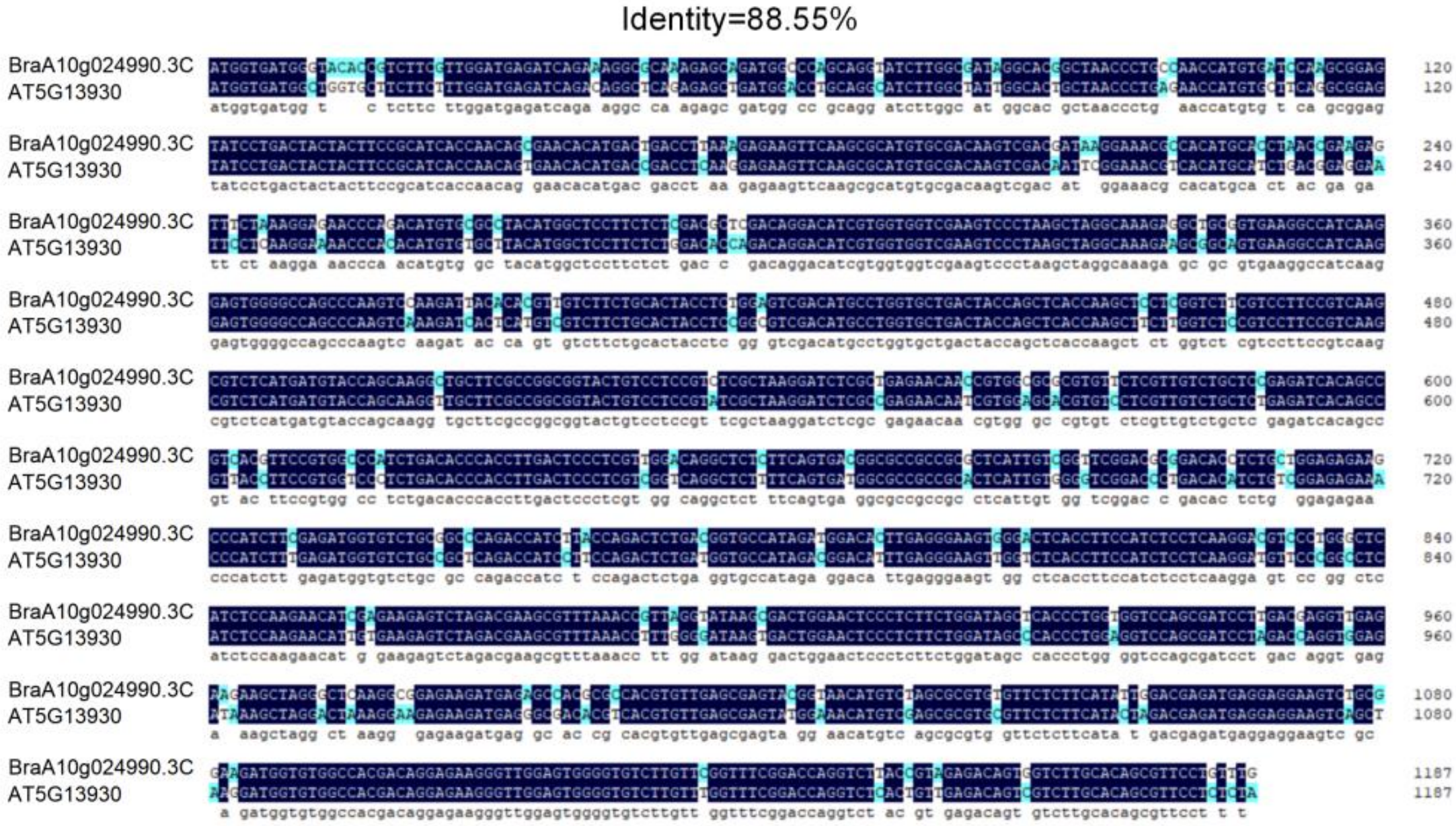

Fig. S12. Amino acid sequence alignment of BraA10g024990.3C (CHS) and its *Arabidopsis thaliana* homolog AT5G13930.

The deduced amino acid sequence of BraA10g024990.3C (CHS) was aligned with that of *Arabidopsis thaliana* CHS (AT5G13930) using DNAMAN. The protein sequences are shown with conserved residues highlighted in black shading. Overall sequence identity between the two proteins is 88.55%.

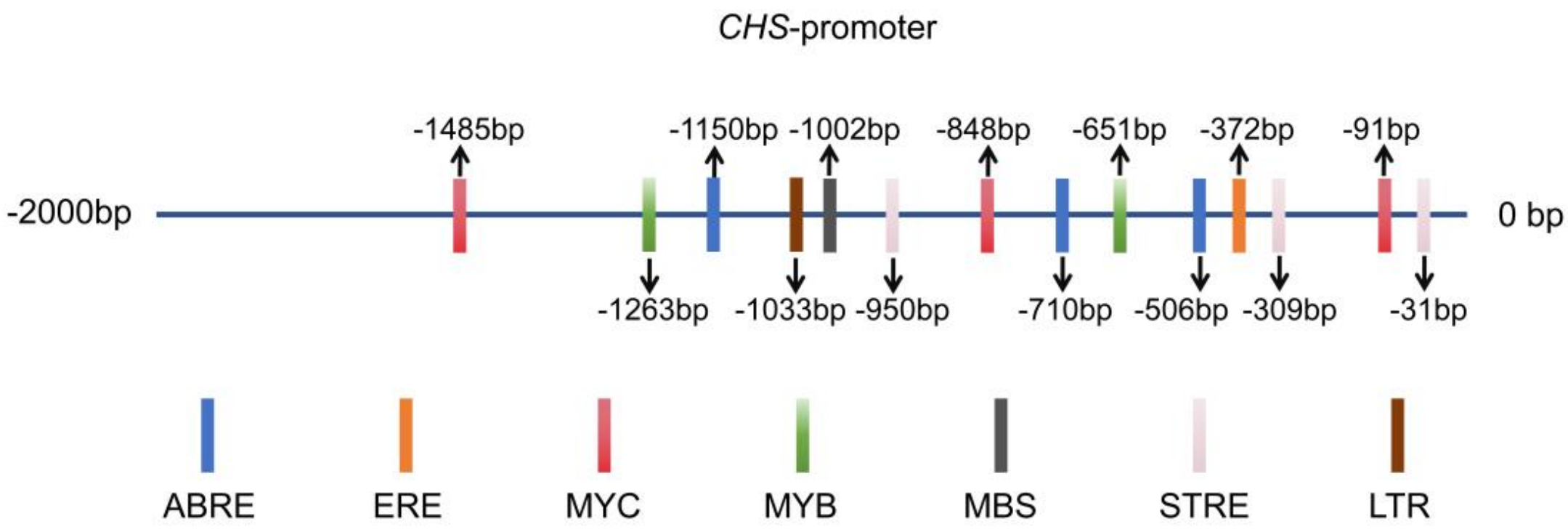

Fig. S13. Cis-acting regulatory elements in the promoter region of *BraA10g024990.3C* (*CHS*).

The 2,000 bp upstream sequence of the *BraA10g024990.3C* (*CHS*) translation start site (ATG) was analyzed for cis-acting regulatory elements. Putative phytohormone responsive motifs, including ABRE (ABA-responsive), ERE (ethylene-responsive) and MYB/MYC-binding sites, are indicated, along with abiotic stress-responsive cis-elements such as MBS (drought-responsive), STRE (stress-responsive), and LTR (low-temperature-responsive). The positions and types of these elements are schematically represented. The analysis was performed using the PlantCARE database.
